# Correction: Chaves et al. Voltage-Gated Proton Channels in the Tree of Life. *Biomolecules* 2023, *13*, 1035

**DOI:** 10.3390/biom15121713

**Published:** 2025-12-09

**Authors:** Gustavo Chaves, Christophe Jardin, Christian Derst, Boris Musset

**Affiliations:** 1 Center of Physiology, Pathophysiology and Biophysics, The Nuremberg Location, Paracelsus Medical University, 90419 Nuremberg, Germany; gustavo.chaves@pmu.ac.at (G.C.); christophe.jardin@pmu.ac.at (C.J.); christian.derst@pmu.ac.at (C.D.); 2 Center of Physiology, Pathophysiology and Biophysics, The Salzburg Location, Paracelsus Medical University, 5020 Salzburg, Austria

In the original publication [1], there was a discrepancy in the citation order of the references, starting from reference [87]. The “Chaves, G.; Jardin, C.; Franzen, A.; Mahorivska, I.; Musset, B.; Derst, C. Proton channels in molluscs: A new bivalvian-specific minimal H_V_4 channel. *FEBS J*. **2023**, *290*, 3436–3447. https://doi.org/10.1111/febs.16751.” was missing from the reference list and has now been added as reference 126.

A correction has been made to Table 1, where “Hcl” has been modified to “hcl” for the cell type THP-1.

**Table 1:** H_V_1 in human tissue and other mammalian tissue.

Respiratory Burst	Cell Type	Function of H_V_1	References
**Yes**	Eosinophil (human) (rodent)	Charge compensation, prevention of cell death.	[10,12,17,36–46]
**Yes**	Neutrophil (human) PLB-985 (hcl) HL-60 (hcl) K-562 (hcl) Neutrophil (rodent)	Charge compensation, migration, granula release, calcium homeostasis, pH homeostasis, ERK activity, phagosomal pH homeostasis.	[6,8,10,12–15,41,45,47–53]
**Yes**	Monocyte (human)	Charge compensation.	[54]
**Yes, small**	Macrophage (human) THP-1 (hcl) Macrophage (mice)	Charge compensation, phagosome acidification.	[52,55,56]
**Yes, small**	Osteoclast (rodent/Leporidae)	pH homeostasis, charge compensation, ROS production.	[57–61]
**Yes, small**	Microglia (rodent) Microglia culture (human) BV-2 (rcl) GM1-R1 (rcl) MLS-9 (rcl)	pH homeostasis, charge compensation, ROS production, microglia-astrocyte communication, neuropathic pain promotion, brain damage enhancing, acidosis exacerbation, M2 polarization reduction, demyelination promotion, white matter injuries promotion, secondary spinal cord damage enhancing, neuroinflammation promotion, pyroptosis increase, motor deficit expansion, autophagy increase, M1 polarization promotion in aged mice.	[22,61–77]
**Yes**	Kupffer cell (mice/rodent)	Glucose metabolism, ROS production suppression, hyperglycaemia, and hyperinsulinemia prevention.	[23]
**No**	Cardiac fibroblast (human)	pH homeostasis, membrane potential, potentially beneficial in ischemia.	[78]
**Yes, small**	Dendritic cells (rodent/human)	TLR9 activation.	[28]
**No**	Sperm cell (human)	Capacitation, acid extrusion.	[32,33,79,80]
**Yes**	Oocyte (human)	pH homeostasis.	[34]
**No**	Type 2 alveolar cells (rodent)	pH regulation.	[5,30,47,81–87]
**No**	Mast cell (mouse)	pH homeostasis.	[88]
**Yes, tiny**	B cells (human) (rodent) LK35.2 (rodent)	B cell receptor signalling, migration and proliferation enhancing (short isoform).	[26,27,89,90]
**No**	T cells (human) Jurkat (human) T cells (rodent)	Apoptosis prevention, pH homeostasis, autoimmune disorders prevention.	[29,89,91–93]
**No**	Cardiomyocytes (canine)	pH homeostasis.	[11]
**No**	SHG-44 glioma cells (human)	Apoptosis prevention.	[18]
**No**	Colorectal cancer (human) SW620 (hcl) HT29 (hcl) LS174T (hcl) Colo205 (hcl)	Prevention of cellular acidosis, support of cancer cell metabolism, pH homeostasis, potential biomarker, and drug target.	[19]
**No**	Basophils (human)	Exocytosis (histamine release), pH homeostasis.	[16,94]
**No**	Ovary cells (Hamster)	pH homeostasis.	[95]
**No**	Breast cancer cells (human primary) MDA-BA-231(hcl) MCF-7 (hcl) MDA-MB-468 (hcl) MDA-MB-453 (hcl) T-47D (hcl) SK-BR-3 (hcl)	Tumor growth, metastasis and invasiveness promotion, (expression predicts prognosis of tumor).	[20,21,96]
**No**	Lung cancer cell A549 (human)	No information.	[97]
**No**	Prostate cancer cell PC-3 (human)	No information.	[97]
**No**	Kidney (human) HEK-293	No information.	[97,98]
**Yes, small**	Nasal epithelium (human primary culture) JME/CF 15 (human) Cystic fibrosis genotype	Airway surface epithelium acidification, proton extrusion.	[99]
**Yes, small**	Ciliated tracheal cells (human)	NADPH oxidase activity driven proton extrusion.	[31,99]
**Yes, small**	lung epithelium fetal (human)	DUOX driven proton release, acid extrusion.	[100]
**Yes, tiny**	Serous gland cell line Calu-3 (human)	Airway surface epithelium acidification, proton extrusion (to a lesser extent than airway epithelium).	[31]
**No**	Skeletal muscle myocyte (human)	pH homeostasis.	[7]
**No**	Glioblastoma cell line (human) T98G	Cell’s survival and migration.	[101]
**No**	Whole heart (rodent)	NOXs transcription and CO_2_ homeostasis control, electrophysiological remodelling.	[102]
**No/** **Y** **es**	Vascular system, Immune system	Atherosclerosis advancement (hypothetical).	[103]
**No/Yes Whole tissue**	Lung (rodent)	Goblet cell hyperplasia prevention. Depression expression of IL-4, IL-5, and IL-13. Reduction of the expression levels of NOX2, NOX4, and DUOX1. Promotion of the expression of SOD2 and catalase. Reduction of the development of allergic asthma through ROS production enhancing.	[104]
**Yes**	Myeloid derived suppressor cells (MDSC) (rodent)	T-cells regulation (via ROS production).	[35]
**No**	epididymal adipose tissue (rodent)	Diet obesity induction.	[24]
**Yes, tiny**	Pancreatic β cells (rodent)	Insulin secretion, ROS production, NOX4 upregulation, glucotoxicity induction.	[25,105,106]

hcl = human cell line; rcl = rodent cell line.

In Table 4, letter designations for “a,b,c,e,c” were incorrectly assigned. These designations have been corrected throughout the table to ensure proper attribution.

**Table 4:** Biophysical properties of some H_V_ channels.

Organism	Species	Channel	Oligomerization?	Selectivity	Gating Charges, *e*_0_	Slope *V*_thres_/*V*_rev_	*V*_thres_ at ΔpH = 0 (mV)	Δ*g*_H_-*V*/ΔpH (mV/pH_o_)	H^+^ Influx at Relevant Physiological pH?	References
Mammals	*H. sapiens*	hH_V_1	confirmed ^f,g,j,k^	>10^6^ P_H+_/P_TMA+_ ^e,i^ >10^6^ P_H+_/P_CH3SO3-_ ^i^ >10^6^ P_H+_/P_Cl-_ ^i^	~5 ^h,δ^ ~6 ^l^	0.82 ^e^ 0.67–0.71 (expressed) ^l^ 0.71 (native) ^l^	13.8 ^e^ −9 to −11 (expressed) ^l^ +27 (native) ^l^	40 ^l^	no (native) yes (if expressed) ^l^	[111] ^e^, [139] ^f^, [140] ^g^, [141] ^h^, [142] ^i^, [41] ^j^, [143] ^k^, [98] ^l^
	*M. musculus*	mH_V_1	confirmed ^n^	>10^7^ P_H+_/P_NMDG+_ >10^7^ P_H+_/P_Na+_ >10^7^ P_H+_/P_K+_	~6 ^m^	0.86 * (expressed) 0.69 ^m^ (expressed)	+10 to +20 −15 (expressed) ^m^	50 40 ^m^	no (native) yes (if expressed) ^m^	[119], [98] ^m^, [144] ^n^
	*R. norvegicus*	RnH_V_1	possibly	>10^7^ P_H+_/P_TMA+_ ^o^ >10^8^ P_D+_/P_TMA+_ ^p^	5.4 ^p^	0.76	+18	44 40 ^o,p^	no	[5], [81] ^o^, [83] ^p^
Fish	*D. rerio*	DrH_V_1	possibly	>10^7^ P_H+_/P_NMDG+_	n.d.	0.69 *	~+10 mV ^ε^	~40 ^ε^	no	[145]
Sea squirt	*C. intestinalis*	CiH_V_1	confirmed ^c^	n.d.	4.4–5.9 (dimer) ^c^ 1.6–2.7 (monomer) ^d^	n.d.	n.d.	~40 ^d^	no	[119], [146] ^c^, [147] ^d^
Insects	*N. phytophila*	NpH_V_1	confirmed ^b^	>10^8^ P_H+_/P_TMA+_ >10^4^ P_H+_/P_Na+_ >10^4^ P_H+_/P_Cl-_	4.7–6.1 ^b^	0.81 ^a^	−3.4 ^a^	47–54 ^a^	no	[121] ^a^, [148] ^b^
	*E. tiaratum*	EtH_V_1	n.d.	>10^6^ P_H+_/P_TMA+_	n.d.	0.77	−23	45	yes	[122]
Mollusks	*C. gigas*	CgH_V_4	possibly	>10^7^ P_H+_/P_TMA+_	n.d.	0.84	−12	49	no ^●^	[126]
	*A. californica*	AcH_V_1	possibly	>10^7^ P_H+_/P_TMA+_ >10^6^ P_H+_/P_Na+_ >10^6^ P_H+_/P_K+_	5.7	0.78	5	43–45	no	[125]
	*A. californica*	AcH_V_2	possibly	>10^7^ P_H+_/P_TMA+_ >10^6^ P_H+_/P_K+_	5.3	0.77	−20	44 40 (pH_i_)	yes	[125]
	*A. californica*	AcH_V_3	possibly ^+^	>10^7^ P_H+_/P_TMA+_	n.d.	n.d.	n.d.	n.d.	yes ^§^	[125]
	*H. trivolvis*	HtH_V_1	possibly	>10^7^ P_H+_/P_TMA+_	5.5	1.03 * 0.26 (pH_i_) *	n.d.	60.0 15.3 (pH_i_)	no	[149]
Corals	*A. millepora*	AmH_V_1	confirmed	>10^7^ P_H+_/P_TMA+_	2 ^λ^	0.86 *	~+10 mV ^θ^	~50 ^θ^	no	[124]
Sea Urchin	*S. purpuratus*	SpH_V_1	confirmed	>10^7^ P_H+_/P_K+_	4.3 (dimer) 1.1 (monomer)	0.69 *	~+10 mV	~40 ^β^	no	[123]
Fungi	*A. oryzae*	AoH_V_1	possibly ^+^	>10^5^ P_H+_/P_TEA+_ ^#^	5	1.40–1.55 *	~−30 (pH 5.5) ^γ^ ~−30 (pH 6.5) ^γ^	80–90	yes	[130]
	*S. luteus*	SlH_V_1	possibly ^+^	>10^5^ P_H+_/P_TEA+_ ^#^	5	1.40–1.55 *	~+20 (pH 5.5) ^γ^ ~+40 (pH 6.5) ^γ^	80–90	no	[130]
Dinoflagellates	*K. veneficum*	kH_V_1	possibly not ^φ^	>10^7^ P_H+_/P_TMA+_ >10^5^ P_H+_/P_Cl-_	n.d.	0.79	−37	46	yes	[133]
	*L. polyedrum*	LpH_V_1	possibly ^+^	>10^9^ P_H+_/P_TMA+_	n.d.	0.69 *	46	40 **	yes ^α^	[134]
Phytoplankton	*E. huxleyi*	EhH_V_1	possibly	>10^6^ P_H+_/P_K+_ >10^6^ P_H+_/P_Cl-_	n.d.	0.69 ^μ^ (expressed)	~+20 mV (expressed) ^μ^	~40 ^Ω^	no	[132]
	*C. pelagicus*	CpH_V_1	possibly	>10^6^ P_H+_/P_K+_ >10^6^ P_H+_/P_Cl-_	n.d.	0.69 ^μ^ (native)	+10 mV (native) ^μ^	~40 ^Ω^	no	[132]

* Calculated from conductance shifts (∆*g*_H_-*V*/∆pH) and *E*_H_ = 58 mV·pH^−1^. ^§^ AcH_V_3 leaks protons at the closed state [125]. ^#^ Calculated from pH = 6.0 and working solution containing 30 mM of TEA^+^ as main cation. ^●^ CgH_V_4 has an activation of −12 mV which permits proton influx at symmetrical pH (physiological) conditions. Nevertheless, the proximity of the offset value to *V*_rev_ makes the inward H^+^ electrochemical gradient small. Thus, H^+^ influx is small at ∆pH = 0 where currents rectify rapidly with depolarization, behaving more like a typical H_V_ proton extruder. During measurements, strong and/or consistent H^+^ influx currents were not detected. ^φ^ kH_V_1 lacks the predicted coiled-coil which is important for H_V_ dimerization, a potential monomeric expression is discussed by the authors [133]. ^+^ Protein sequence analysis predicts a C-terminal coiled-coil domain. ** LpH_V_1 pH-dependent gating saturates above pH_o_ 8.0 [134] similar to rat, human, *Karlodinium*, and *Emiliania* H_V_ channels [137]. ^α^ Inward H^+^ currents are onset at large inward pH gradients (1–3 ∆pH units) [134]. ^β^ estimated from *g*_H_-*V* curves shift between pH_o_ 6.5 and 7.0 (~20 mV), at pH_i_ = 7.0 (Figure 2; [123]). The *g*_H_-*V* shifts between pH_o_ 6.0 to 6.5 and 6.5 to 7.0 are not identical due to experimental limitations reported by the authors. The calculated value on pH_o_-dependent gating agrees with Δ*g*_H_-*V*/pH_i_ unit (Figure S1; [123]). ^γ^ from normalized *g*_H_-*V* curves (Figure 3; [130]). ^δ^ from Q_on_ of dimeric W207A-N214R mutant ([141]). ^θ^ from *V*_thres_-ΔpH and slope of *V*_0.5_-ΔpH curves (Figure 6; [124]). ^λ^ apparent from steepness of normalized *g*_H_-*V* (Figure 6; [124]). ^Ω^ from current densities (Figure 1; [132]). ^μ^ the slope *V*_thres_/*V*_rev_ was calculated from shifts of *I*_H_ onsets and *E*_H_. *V*_offset_ was estimated shifts of *I*_H_ activation, *E*_H_, and Equation (3) (Figure 4; [132]). P_H+_ = proton permeability, P_TMA+_ = tetramethylammonium permeability, P_TEA+_ = tetraethylammonium permeability, P_K+_ = potassium permeability, P_Na+_ = sodium permeability, P_Cl-_ = chloride permeability, P_NMDG+_ = N-methyl-d-glucamine permeability, P_CH3SO3-_= methanesulfonate permeability, n.d. = no data, *V*_thres_ = threshold potential, *V*_rev_ = reversal potential, ∆pH = proton gradient (pH_outside_ – pH_inside_), *g*_H_-*V* = proton conductance – voltage relationship, pH_o_ = external pH, pH_i_ = internal pH.

Additionally, several textual clarifications have been made: the term “putatively” was added to qualify the described mechanism, the phrase “translate to” was introduced to improve the accuracy of the affected sentence, and the repeated year “2006” in the description of the Ciona intestinalis homolog was removed.

With this correction, the order of some references has been adjusted accordingly. The authors state that the scientific conclusions are unaffected. This correction was approved by the Academic Editor. The original publication has also been updated.
