# Supplementary material for: Voltage-Gated Proton Channels in the Tree of Life
Source: Biomolecules. 2023 Jun 24;13(7):1035. doi: 10.3390/biom13071035 (PMC10377628; doi:10.3390/biom13071035)
Supplement: Supplementary file 1 [file biomolecules-13-01035-s001.zip › biomolecules-2423764-supplementary.pdf]

Supplementary material

Figure S1: Tree of life

Opistthokonta – Animalia – Vertebrata

Eukaryota; Metazoa; Chordata; Craniata; Vertebrata; Euteleostomi; Mammalia; Eutheria; Euarchontoglires; Primates; Haplorrhini; Catarrhini; Hominidae; Homo.

Homo sapiens hydrogen voltage gated channel 1 (HVCN1), transcript variant 1, mRNA. NM_001040107

MATWDEKAVTRRAKVAPAERMSKFLRHFTVVGDDYHAWNINYKKWENEEE
EEEEEQPPPTPVSGEEGRAAAPDVAPAPGPAPRAPLDFRGMLRKLFSSHR
FQVIIICLVVLDALLVLAELILDLKIIQPDKNNYAAMVFHYMSITILVFF
MMEIIFKLFVFRLEFFHHKFEILDAVVVVVSFILDIVLLFQEHQFEALGL
LILLRLWRVARIINGIIISVKTRSERQLLRLKQMNVQLAAKIQHLEFSCS
EKEQEIERLNKLLRQHGLLGEVN-

voltage-gated hydrogen channel 1 [Xenopus laevis]. XP_018099695

MAGCLRHFTSVGDDTKKREWKEEDVEVAHEEEKKNTPHPFIASYSLRGAL
KWLFSSHKFQIVIISLVILDALFVLVEVLLDLELLAEKVDHIIPEIFHYL
SVSVLSFFILEIAGKLYAFRLEFFHHKFEVFDAAIVVISFIIDIVYISRE
DIFNAVGLLILLRLWRVARIVNGVIVSVKSRAEEKIHKLEENQRSLLEKV
TQLEQQSAQQEQEIARLQKLLKQHNVFPDS-

voltage-gated hydrogen channel 1 isoform X1 [Xenopus laevis]. XP_018104580

MAGCLRHFTSVGDDTKKREWKQEDVEVAYEEPLKNTPHPFIASYSFRGAL
KWLLSSHKFQIVIICLVILDALFVLVEVLLDLELLAEKVDHIIPEIFHYL
SISVLTFFILEIAGKLYAFRLEFFHHKFEVFDAAIVVISFIIDIVYISRE
DIFNAVGLLILLRLWRVARIVNGVIVSVKTRAEEKMHKLKEQKGSLLEKV
AQLEQQCAQQEQEIGRLHKLLQEHNVFPAS-

Pan troglodytes XM_016924207
Macaca mulatta XM_028829869
Balaenoptera musculus XM_036823351
Mus musculus NM_028752
Rattus norvegicus XM_017598517
Danio rerio NM_001002346
Lacerta agilis XM_033159246
Gekko japonicus XM_015415738
Microcaecilia unicolor XM_030220075
Petromyzon marinus XM_032947247

Opisthokonta - Chordata - Tunicata

Eukaryota; Metazoa; Chordata; Tunicata; Ascidiacea; Phlebobranchia; Cionidae; Ciona.

Ciona intestinalis voltage-gated hydrogen channel 1 (hvcn1), mRNA. NM_001078469

MEGDNCNKSRHKSHNMINPNYASVRCTQPLPSVIQLRSRNKMIGITEDPS
SDSEPVSSNQPLLLTNLSYEVHTFNDNNNHERPAPQEQSTQNTMISMQSE
QKSDRFTASNLGMFQYMKFEIGEDGDDHEEEAILTNREKLRHILHSKPIH
VAIIVLVVLDSFLVVGELLIDLKVIIVPHGNPAPEILHGFSLSILSIFMV
EIALKIIADHRHFIHHKVEVLDAVVVVISFGVDIALIFVGESEALAAIGL
LVILRLWRVFRIINGIIVTVKTKADDRVHEIKKKNSELELQIHNLEEKLS
QKEQDMSRLHEILRCNNIDIPPTVPLTTSVQIHSTTTASADV-

Ciona intestinalis voltage-sensor containing phosphatase (vsp), mRNA. NM_001033826

MEGFDGSDFSPPADLVGVDGAVMRNVVDVTINGDVTAPPKAAPRKSESVK
KVHWNDVDQGPSEKPETRQEERIDIPEISGLWWGENEHGVDDGRMEIPTT
GVGRVQFRVRAVIDHLGMRVFGVFLIFLDIILMIIDLSLPGKSESSQSFY
DGMALALSCYFMLDLGLRIFAYGPKNFFTNPWEVADGLIIVVTFVVTIFY
TVLDEYVQETGADGLGRLVVLARLLRVVRLARIFYSHQQMKASSRRTISQ
NKRRYRKDGFDLDLTYVTDHVIAMSFPSSGRQSLFRNPIGEVSRFFKTKH
PDKFRIYNLCSERGYDETKFDNHVYRVMIDDHNVPTLVDLLKFIDDAKVW
MTSDPDHVIAIHCKGGKGRTGTLVSSWLLEDGKFDTAKEALEYFGSRRTD
FEVGDVFQGVETASQIRYVGYFEKIKKNYGGQLPPMKKLKVTGVTITAIQ
GVGRGNGSDLSMQIVSERQEVLLCKFAEGYNCALQYDATDDCVTCEVKNC
PVLAGDIKVRFMSTSKSLPRGYDNCPFYFWFNTSLVEGDHVTLKREEIDN
PHKKKTWKIYRDNFTVKLTFSDAEDI-

Polycarpa mytiligera GIUT01151021
Salpa thompsoni GFCC01108773
Oikopleura dioica GCJN01019160
Phallusia mammillata LR785897
Aplidium turbinatum OU964926
Ascidia mentula OX387201
Styela clava XM_039415523
Clavelina lepadiformis OX392441

Oikopleura dioica comp23289_c0_seq2 transcribed RNA sequence. ACCESSION GCJN01027812

MSENSSVVESTPFLEILKSDMTCWEKTSRVVHKLLHSHTTQAILLFLVLV
DCAVIACEIVFDEKVKTHNDYCGANSTLCQEEIDNEQTKKWKKIYKALYY
TSLTLLSVFVVEFILKIVFSAKKFLKSWVHIFDALIVISSWILMLIMLNK
DVKNGMIAEFLIAFRVIRLIHGMKEALEEANELLHENMEEEIHALKAENN
KLKEELLAVKDR-

Oikopleura dioica TRINITY_DN2869_c0_g1_i1, transcribed RNA sequence. GKAI01006710

MDNRISKFFSSDTEAYLERTPVTISEKIAHFLHSSTCHLIVIVLVLVDCA
FVLTELIFDGKLDTYAEYCDPTPSCSEQSHKLHHWEHVTHVIHICSIIVL
TIFMIEVILKVIYTREHFFSHKIEMIDGVVVLLSWILDLIMLNPNLVGKW
AEFIIIFRFLRLINGMILSAKQAADKRVHEQKRKVQELEKELVDLKKQYE
TQNNQ-

Opistthokonta – Animalia – Cephalochordata

Eukaryota; Metazoa; Chordata; Cephalochordata; Leptocardii; Amphioxiformes; Branchiostomidae; Branchiostoma.

Branchiostoma belcheri voltage-gated hydrogen channel 1-like (LOC109464640), transcript variant X1, misc_RNA. XR_002139895

MDKLKNAFKFELFQNDDSSSVITSSDATSSSGNAEPKTFREKLLHVLHST
EFQVAVVILVIVDCILVVFELLIDLGGIKLCEEAVRAECESAGTTATMTP
AEEAEKEAECDHPAPEILHYMSIAILTIFLIEIMFKVYAYQKDYLKHKME
LFDAVVVIISFCFDVAYANHEDAFDGIGLLVVLRLWRVTRIINGILMSVQ
HTAEKKINAHKQARQEVQEELNKMIAHAHDLEKEIDLLRKTLRENGISVE
SIPRTPEVSSSQVKVEAEITPTTEYATPAHFSAFSGQDDTQA-

Branchiostoma belcheri uncharacterized LOC109463761 (LOC109463761), mRNA. XM_019760615

MTPCDGEKKPPPSGEATVGGIFPDEIAVIKKQAQNDKRRPLKIVIIAGVA
VMAVLAIGVLLLITRPSRHGVVTCSLNFWTGKQLLHETVETDKDAETDAF
YTEGSRGEAAVMYDHSSMMKAFKLSRSNKTCFIFEETQGEKNAVKKTAEE
LEEKQDGSLQFAEYGGAMLMTVDTERPARPVLSQKLQNFCGQLEPRWAKL
TPATEEEQQGDRVEIIMPAEARDMGELAEMPVGPGNEKETPPTPSSTHPL
TESRDHHHDNQDCRHKLKHMLERQSVHIAIVVLIVLDTLIVIMELLIDVR
VIKLCPDPPDVCVPKAGHNGTTGLVTTGAPGHHVIDAGDHGTGGHEECHH
VLIEVLHVVSILILCIFVVEIALKIYVDRLEFFKNGFHVLDAVVVLVSLG
LDIASLVRPSAFTDAGGLLILLRLWRITRIVNGIIISVEEEWEHKVNHLK
HEHQLVERERDRLLKENALLQKTLTNHGIDIPKLPPDSGDESTCEFEFE-

Branchiostoma belcheri uncharacterized LOC109467017 (LOC109467017), transcript variant X1, mRNA. XM_019764911

MPRFKGQQQHHETCRHLAVEDVMVADELGLGMSSADLYNRNVEGDFLDLE
AAIFCDIEEEKTCKQRVQQLLDGPATQITIVLTSWLLSHVLLLELLVDLS
AIHFHHDKMVARIIHWVGLGVLSVFTVEVLTRLVCHQMQFFDKKIEVLDL
AVVIIACVPMIVVSVELAPSTAWDGFSLVIILRIWRCYRVVQGCVSPVRE
EASRKVHVLLQAQRRAHQELQTLYLMHDENQEEIHRLRLLLGRREAEEDN
VSQQLQVALERKDSQYVAQLIHTIEQRQGRNKGRQRDGGRDSPYSTIVVH
AHQLSDSNDVPSGTIASRLSESTTGDSGICEETRPQQQTNDESRNHANSS
GVTQLPKCGQQALTVVEPRGSHQQHSNRSPCSNSKRNPAVSSCDMDRIDV
IGSEIILQHRNDSTGKKFLDGKLPSNKDSRLLFRDNKSMPSNGKVSHSKN
KVSEIKVNKSLDDDHKKAVSMDNKTVSKNNTATAQTNGKVRKGKKKAGSG
DSYVNGALLLEMTELQQKGDTGYCNEIVIEQFYQNGNAPATAL-

Asymmetron lucayanum GETC01094801 GETC01046531
Branchiostoma lanceolatum JT904261
Branchiostoma floridae GESZ01013291

Opistthokonta - Animalia - Hemichordata

Eukaryota; Metazoa; Hemichordata; Enteropneusta; Harrimaniidae; Saccoglossus.

Saccoglossus kowalevskii voltage-gated hydrogen channel 1-like (LOC100367669), mRNA. XM_002734179

MWNFSKIHVPYIRNSCDCFSYHHHYHKMDGFGFKRMQESKQDGQLRVITK
DDTSDSLASDSQDEATKKEFNGFRGTCLAVMNHHYCQVAIVVLVILDVIF
VLAALVLDMQMSTPSDCGNSTETQEFEENTASAVLHYCSLTILSLFMIEI
SLRIYCMRLEFFKHKLEVFDAVIVIISFSFDVAYAISPDTFHDILGLLVI
FRLWRVVRIFNGVLISVKNQNEKKLAAQRRKCSELEQELEKFRQYCTVQE
NEIELLRDELKKHGITLEAEEKKERPQSLTQVDVIVEVNKVNEKTIESLD
RKDFKEDIDAVDYSDVTPDPPSTETDKSPTRSTSSTIPESPQEPDYGSNC-

Saccoglossus kowalevskii voltage-gated hydrogen channel 1-like (LOC102802600), mRNA. XM_006812030

MAADEESHQGPVTIVRDGFENRSSTPRSFSTPDLITATHRRRPRPKYCMA
MERFINSVIVQVIILLMVVLICLIATTETLVAYRRLKFDSSKTAETVITV
LHYVCLFFFIVFVFEVIFRICAMGVEYFHQPMQIVDGLVVFLTFTLDVSL
WLAPVSHPALHTLSFLIIIRMGRLHLIIKGITDRVREDADVQIELERIMR
RNIEACADKLQQQCDQQNKEIAFLKDLLQQHHIESLQSNGSVIHTDSETL
KKAESSSSSTAGTIDGSPIDKRKCSVDNIAVTNDHYATATSTAILQSAMD
DVINRSQNIENIEKDKDEVKTSQDNQNITIIEETPATPLKECKAVSVSMP
QIGTAANPPIRYEDLDTLNLELAEIRRLSQEALLQDFTTPLEGINNNNIE
SIAIE-

Ptychodera flava GDGM01225783

Opistthokonta - Animalia - Arthropoda - Insecta
Eukaryota; Metazoa; Ecdysozoa; Arthropoda; Hexapoda; Insecta;
Zygentoma; Nicoletiidae; Nicoletia.

Nicoletia phytophila proton channel mRNA, complete cds.
KT780722

MWLKMDAHKRLSEDLEKVIMKEDGNSSIMTEPDHNIQPSKTVRERLRKLL
HSHKFQISVITLVIIDCLLVITELLIDLEMHEEESLAQHVLHYCSITILS
IFIVEIFLKLYAFRQEFFKHRLEVFDAIIVIVSFALDIAFRNSRDALSGV
GLIIILRLWRVARVLNGVVLSVKMQAEHQLEREKQRGMALEGELSRCRQV
CAAQQRELDVLRAVLQHHGLDQQLPDGNRVDVVADVEKR-

Extatosoma tiaratum GAWG01024136
Aposthonia japonica GAWU01255994
Peruphasma schultei GAWJ01019192
Aretaon asperrimus GAWC01068486
Medauroidea extradentata GAWD01057497
Ramulus artemis GAWE01048256
Sipyloidea sipylus GAWF01047109
Clitarchus hookeri GFVY01085196

Opistthokonta - Animalia - Arthropoda - Chelicerata
Eukaryota; Metazoa; Ecdysozoa; Arthropoda; Chelicerata; Merostomata; Xiphosura; Limulidae; Limulus.

Limulus polyphemus voltage-gated hydrogen channel 1-like (LOC106464594), transcript variant X1, mRNA. XM_013924749

MEDTTEKTGDSTSIATNLESDPDIQPLITFRERLTKLLHSYKFQVGVITL
VIVDCLLVISELLVDLNILSVNSHSSAPHVLHYLSISILSLFIIEIGAKL
YAFRLEFFHHKLELFDAVIVLVSFALDITFRDKESAVSGVGLLIILRLWR
VARVLNGIVLSVKTQADHKLAKEQKKRENLEQELARSRDYIAALEEEVET
LRRILKDNNIKELPPTVIDNGAFKCTTLNVVAEVNHMIT-

Limulus polyphemus transmembrane protein 266-like (LOC106463214), mRNA. XM_022390662

MRNSDPLVWGLTGDTSLDDDGPTRLCEKIWATVNGKVFSAVIVTLVFVEG
LVIFSELLIDFEVVQDPQWKTSLNCSANVSGLVFSSAGANSPKLRMAKDI
LSYISIVILIIFVFEVGFRLVSGRARYLIQGMEICDAIVVLVAFGLDIAF
LTTPSKKGAGKEAAVLIILLRLWRIKRILQSVIDNTRLEMGHFLSICERE
KIQAEHKVDILILKVEDLEHEVAYLKEKLKKTEKESLYAKRQRKKDGYSS
TQHKHPTITIGVETSPARHPCTGTQTAIVICEQHIPEEKVTKEQVMDMRT
FADVTSTRIIADALCMVTGNPNQFLRSPTTGGSTVKDGTTSHCRIAGDFE
SGYISNVSGITWDKAASRIPTLGTRTTLKCPESSPESGYGSSSSARNPAA
SSVSPLDTGTETASTSTGSSKHTDTVFLFPDPTGQRREVELGVEMDILEE
ISEIERVKHIEFDPNKQDQDIPMTSL-

Limulus polyphemus uncharacterized LOC111087200 (LOC111087200), transcript variant X1, mRNA. XM_022393049

MRNFSMFFNSKEELMRNSEPLVWSLTGDESLDDDDSTRLCERIWVSVNGK
VFNAVIVTMVFVEGLVVFSELLIDFEIVQDPHWKTIRNCSANVPALIFPS
SEAVSPNLRNTKDVLSYISIVILIIFVFEIGCRLAVGRAKYLIQGIEICD
AAVVLVAFGLDIAFLTTPSKKGAGKEAAVLIILLRLWRIKRVLQSVIDKT
RVEMGHFLSICEREKAQAEQKVDILILKVEDLEHEVAYLKEKLKKSEKES
LCAKRQRKKEGYSGIQQKHPKITVGVETSPARHPCTGTQTARAICEGFIL
DEKATKRKVMDITTFAEITATRIIAKALCVASPDSGQYVRFLATKDAVGI
SGTTLCRRIDGSFESGYGSNTSGMTLDKTTNCIPSVGIRRTVKCPESSPE
SGYGSSSSARNTVATSVSPLDIEMETTSVSTGSTKQTDTVFLFPDSANQC
RKAERGVEMDILEELSEIERIKQVEFDPNKQDEDIPMTSL-

Parasteatoda tepidariorum XR_001584685 XM_043048148
Dermacentor andersoni XM_050188908 XM_050168704
Ixodes scapularis XM_029987868 XM_042290414
Varroa destructor XM_022812381 XR_002674557
Metaseiulus occidentalis XM_003738312
Tachypleus gigas GILM01000651
Carcinoscorpius rotundicauda GILQ01010360
Piratula clercki IBTM01003006
Pholcus opilionoides IALF01028455
Zoropsis spinimana ICQH01009853

Opistthokonta – Animalia – Arthropoda – Myriapoda
Eukaryota; Metazoa; Ecdysozoa; Arthropoda; Myriapoda; Chilopoda; Pleurostigmophora; Scolopendromorpha; Cryptopidae; Scolopocryptops.

Scolopocryptops rubiginosus strain wildtype
C96881_a_4_0_l_1490, transcribed RNA sequence. GCIY01020623

MENHHKMSDDLERVIMKDDSSSSVVTEEDPQLQQFQTFRERLAHLLHSYK
FQVAIVGLVILDCLLVIGELLIDLRILEIQEHDYGAGEADLSICHQVLHY
MGITILSMFVVEIIAKLYAFRLEFFHHKMEIFDAVVIIVSLSLDLAFLDK
HNALNGAELIIILRLWRTTRILNGIVISVKTQAEHRLHRERRIKEALEQE
LAKYREYCTEQEQEIEALRGLLRKHGISENEIIESPMTLINQMDVIAEVN
HTNEQNDKLIA-

Scolopocryptops rubiginosus strain wildtype
s6321_L_19063_0_a_4_4_l_1908, transcribed RNA sequence. GCIY01027533

<YFSLTILSIFIIEMLLRIVSGKVQFFKQCMELFDAMVVFAAFSLALSFL
TTPKDGRNVAIFVVILRLWRVKLIVHSVIHDTKRQMSHIINAYKRDKIQA
EHKVELLILKVEDLEHEVAYLKEKLKKTDKEAAQRKKHLRVSSQLAEFPI
TPQIEKYNPLFHNPILSQRPPPEGRTESPPRDQDVERLERFAECTVKAIL
RTASNELISKDRWQQKRQSQRRRSAGASDDIHEASSSSSNIIGASSSPTE
LRQLTDTDGAEGLLSNNSAACFRVAQSSQEELDELSEIERIKKIRFDPTK
EEKNIPVTAL-

Henia illyrica GESB01025859 GESB01004335
Schendyla carniolensis GESL01004004
Scolopendra cingulata GCAP01021964 GCAP01017169
Lithobius forficatus GCAY01038678
Cryptops iheringi GJOG01009205 GJOG01023410
Strigamia acuminata GESK01000419
Scutigera coleoptrata GCAQ01037316 GCAQ01012485
Polydesmus complanatus GESI01001010
Himantarium gabrielis GCIL01017378 GCIL01018683

Opistthokonta - Animalia - Arthropoda - Crustacea
Eukaryota; Metazoa; Ecdysozoa; Arthropoda; Crustacea; Branchiopoda; Diplostraca; Cladocera; Anomopoda; Daphniidae; Daphnia.

Daphnia pulex voltage-gated hydrogen channel 1-like(LOC124207115), mRNA. XM_046604416

MESTKEMLLPRPEVDGIQQSQIEMVTVGQDPGMRLMNDAQVDQQRDALLS
KDIVLHPSHVYASDPNLAMMPDSSSRARLRRILSSHRFQVFVVSFVIVDC
MVVIAELLMDLRILGMMEEYGMKNRNKVSLEAHYIVPDVLHSISIAILAM
FLLETVIKIAAFGLSFLRMGWEIFDTVVICVTFVLDVLMQHSHSSTNGLG
LFIILRLWRVARILNGMVRSVRSQAVRHVECEKRRREVLEDELLKYRELC
QRQKKLLAEMENLLKNHNIPLPDNLVMLPSP-

Amphibalanus amphitrite XM_043361853
Pollicipes pollicipes XM_037237188
Homarus americanus XM_042376150
Eriocheir sinensis XM_050860406
Cherax quadricarinatus XM_053773827
Procambarus clarkii XM_045762000
Penaeus vannamei XM_027375813

Opisthokonta - Animalia - Nematoda
Eukaryota; Metazoa; Ecdysozoa; Nematoda; Chromadorea; Desmodorida; Desmodorina; Desmodoroidea; Desmodoridae; Stilbonematinae; Laxus.

Laxus oneistus TRINITY_DN84728_c0_g1_i3_9dedup, transcribed RNA sequence. GJNO01007626

MTGHVYKKKRPLSAHAAVGGAGREEECVIMNTEGDGSSSVTSESEHGNEH
QPPRILHGRSLREWLDAVLHSTKFQVLIVCLVIFNCLVVIVGLLIDLKIF
ALERKNNVAAEILRYVGIVIVSLFVLEMALKVLVLGKRFFKHKMEMFDAV
VVILAFSLGLSFGGRENSADGFGLLIMLRLWRVSKILNSMITSVRKEAER
KMAKERRSRRALEREVAKLREYCLQQECELQMYRLLLQQNNIPEPTVLRP
PPAPRTLSVIAEVNELDPPGTATFLAPLSAPQSGTDDSSTNSGSFEGPLC
SDRPPANG-

Trichinella spiralis GEBN01052808
Plectus murrayi GGJS01022439
Koerneria luziae GIUA01076674
Trichinella patagoniensis GECA01018136
Trichinella zimbabwensis GEBX01035233
Trichinella papuae GEBW01009651
Trichinella pseudospiralis GEBS01033974
Trichinella britovi GEBO01040153

Opisthokonta - Ecdysozoa - Priapulida
Eukaryota; Metazoa; Ecdysozoa; Scalidophora; Priapulida; Priapulimorpha; Priapulimorphida; Priapulidae; Priapulus.

Priapulus caudatus voltage-gated hydrogen channel 1-like (LOC106809460), mRNA. XM_014812541

MKGLGVQGFKKVSEDLEKVIVRDDATSMSTVESEEDNFRTRMPLHDRVNA
LIHGQRFQIFIVVLVIIDVLLVIAELLVDLKVFEMEPGDSGEDASESAIG
EVLHYASLAILSLFMVEIVVKLYAMRLSFFKHKLEMFDAVVVVVAFSLDI
AFTTNKGGAVNGLNLLVILRLWRIARIVNGIILSMTAQAEKRLHREKRER
EAVEDELGKFREFCARQTQEIERLRDLLELNGISAHKVERTAFGSQLQVV
AEVNDIITSKKLEADT-

Opisthokonta – Animalia – Lophotrochozoa - Mollusca
Eukaryota; Metazoa; Spiralia; Lophotrochozoa; Mollusca; Gastropoda; Heterobranchia; Euthyneura; Tectipleura; Aplysiida; Aplysioidea; Aplysiidae; Aplysia.

Aplysia californica voltage-gated hydrogen channel 1-like (LOC101848758) XM_005100609

MKLDGLRKMQDDLVKVIERDDTSTVTSDSDETIARGPKTLRETLDDVIHS
QKFMVFIIVLVVLDCLMVIAELLFDLEIVKLGEEHHYIPKIFHYGSLGIL
SLFLIEIGLRIFVLRLDFFKHKLELFDAVVVIVSFILDIVFRDNEDAATG
VGLLIILRLWRVTRIVNGIVLSVQKQAEKKIEREKHLREECEQELAKFRE
YCMAQEAEIEVLQGLLHKHNIEFTTNKITRPESRVQVDVVAEVNSMTAVA
ETDIPLSPSQPGEQEISLSSGDNVTDAVVVV-

Aplysia californica uncharacterized LOC101850633_X1-X4 XM_005093050

MRMSRSIEYPSEKNGEPSCIEAEQRSGETKMLKSEQSQDEAETSDWSENE
DSHSGKLDANSCKGKLAAFLKTNLVQYSIIALVILDCLIIVMELLIDMNI
IVFPEDDP*HH*PPGEGSS*HH*PVAFASRSSNLTGDN*H*TVYPA*HH*I*H*T*HH*DNS
SNLTMYGNDSA*H*AAPV*HHH*TNKEKAEHVLHALSLTILSIFMVEVCVKIYV
EGKHMLKQKAEVFDAIVVIVSFTLDITFSFVSVSKAASEAAGLMVILRLW
RVTRIINGVIMSVKLDANKKMEVHKKARRKLERENKRLQAKIERLEREVA
TLKQKMATSSTPQMSFEMQSGLSVERSPSGEMRENSAQV-

Aplysia californica uncharacterized LOC101855857 XM_005094218

MGDAEPRAHPRPRGQASPFMPRLRKRGEKLLHSKYVVILVIILTVTDCAL
VIAELILDLSSVKKTQGATEAMTLSFVEKIIKKYPDEVAPLHSLTDVFEE
LNHADIVWNNTRNSRGHDLDPDLERNLHHHHHKNRDALHTSVTTPTSVPV
VGGESVAEGWPLSSDTPLGVGNFSRALRTLWIQKRNYSSNSRFRRSNRND
SLRDSSAVLSRLLERTRLEIEKVLSKLSRRRKRSSEETGGLTAEYEATDS
GEAGDTSRNSQELEDEVLNSDFFENNYGKNNGKTYLSSLTGLIVKILTMQ
SNETGPILGGPARYAEAPLTVESAGKGEGKGAKHVSDQDILHKYRLEFHH
SEDMEIAHKLHYASVAVVSILLIEVTMKIICAGSHFLKRKIEVFDAVIVV
ASVIVDLIFIKGLNQFPVDDSIFVLAFLLPWRVIRVVNSLVMAVIDHEHV
KLRLLYSRKKKLDKTVETLRNEVDELKGMMQDIRQFCIKEGIEASRIDSL
LGKFAPRRRKDSKFYTLVKLVMSTASINNNNNDNDSVSSSSMENDLRDYA
NRDSVLNEATSNENTVTSLKQYLSVPFFSGGNRSNTLDIESRGSGRSGGS
PSIYITSPASDDEAPVFSFDIADEDDVDMSNDQDDAGSQDDETSIQAGSD
AATIAVTSAEVNTVSPNVAFYVGSQSSLCSVHSQEDIRTVVDTNDNYERN
FPMCGVPCASGGEDLAAAALDDVISNSPTVNSNSWGPSRHYSRFLTVPCC
TLSLSAVTNTTSTATSYPSSSNNTNNNSNSNNNNNNACAETHPLLGDPPP
GQNMTRSVSDNCDVTATQYGARTCGGRYGSPVPMRRKPCLSEKRRSYARA
RSESIENQEFIPLMSQGANKGRVRHHSDLEGRPSTRKDDLKRSRSHSPSP
MVLLGVPGQTKKYSDPPPSYQAASRSMNDVSSAGKGQSGNPHANNLGKDG
RILRRSCLSLTSEGRKRRGKSPQRVSFKVS-

Aplysia californica uncharacterized LOC101862058 XM_013086351

MRQDVFTITPSTKAHGTQQVPPSPGGILKTPGKVSCNPNGKFRPGPQGAG
MRRSVSVESTSTFDPSELERPGNLSSRQRCQRRLSVLLHTHVVLILVCTL
AALDAVCVIGQLICDILIMREKLDHFEVIDDQLTDILFDHIPKLNQSLHP
KWNLDAILDVLTGRDHHSNDGPVPTPAPLASNLSSLMSVVNSSVLQNFTH
MNQSALHHRVRRAAKQEVPGHEVDHGLLYDLTHTFHLGSMVILSLLLLET
LLKVFAMGKKLRHHKLEVFDAVVVAISWALDVAFWEGIWAHPGTTAATIL
IYMLPWRVVRIVNSFVLVIQEKDHVQLKIVKQRLRQSLKKSKEFTDKASS
YRHEVKALAGLCRKLGANESEITACSPSGKACRRGSIHSVLERAASLTFI
STLSSMGSLPSLFDMGEMSSDEEDSRPQHQDLDRTTSQAPTLKSAFSSTT
LDSGSVVLSIDNDTGGGMEHPVFDDASNSSTKSSDVKHSADKGKREVERT
SSSDSAPPSYHIAVSKTDSNTRL-

Aplysia californica uncharacterized LOC101857936 XM_013080089

MRKITITSSKMPYRKKSEIPKAWSMETLPPPGMVTSRPLRKKGLFHTQTC
MEGSWQVLAAQTLKIASINGLGELDKLENELEDEIRYENRSPSKTAVGRL
RRQGQHQLHSKVVLLIVVVLNVIDCLLVMAELTLDFHHVSHRLQNKLDML
ESFIYNMITKHAPVLDNIPRSPRKSNVLLQKILDANVVWDTSQPNVTNFA
SNCAHLLKSTAENASQSLTGYMSNMLNNSDWSEPNACFQPYMAAQNTTVN
SAAANQKYMLSTDGQTNEKLTIVAHKLHYISISILSVLVVILLLKMICSG
KRFFRSRMQVFDGIVIIISFILDLVFIEGVTILKMDDFVLILTFLLPWRI
LRVLNSLIVAVLDKQRLNLKIIYTQKKKISRNLSEVNNKMEVMQRHIEVL
QNLCSSRGLADGDVKKVLGRELSTASAKSGSSQKNGSSGGLAGMMALGKL
AFQAADAFAPITQGSRKSHTPKTTKQNKPALNGSAPNLLTPTEEDPPTPP
HLAHSMSQPQGESNTTSSNSHVNTVAADIEDTSRPSFTLHNETGSAEGTE
SGIGGSVGGGIDGSGVDDKTADKENTPPTPTPIPTHDTYLDIESQPNANT
GLETSDSSTGVNTNVATTTTSPDSVTVQIANGSGPPMDLDSNSNEERKTS
VSLPDFSDDIHLEDSDVV**S**RL-

Aplysia californica uncharacterized LOC101852977 XM_013090418

MSPGILKGPSTPRVDRPPVTISTSVRLSAQHGHRNGVGKQARMSTGLLTG
CRQRTLKCINCRPFLIAICALVVVECACVLAELMVDLQGIKFRFENEELE
IKRFVLHLRTKYPGAFSDDRARTMTDVINLLDQAIVLRTRQDLLTPPKAP
CFCPCANETSGPAAALRNSMSPAGSKQMKSHGKVQPLGVVDGGDLSRVPR
QKAQPDKYDKTRQEMIFTTPNVELTHTQIDITSSSSSSSSSSSSSSSSSS
SSMSSPPSSALSSKLTTAQAGDVTTGAHDFGQAGSESRFRRSSGNHDAPK
METVNTSGSKQMLLHSRVASFSLNTPPPGDPLLRLNWKGNHPVLSNTSEQ
SSGKSQFVFSFSQKIHRALREVGLQRLALGRRLMGVAIENDQPTPAGHPI
SNVISTHLPNHDAMTTTEAEAVTISSPSLQSGFSAHKSTADPVKGWVQQT
TKDTVLGAITPESDSEDSLQNHNEGSSPSDVPTRTSVTDPPASPAPISKR
ATGTLTPDSQVFTKREDDIQNSNMKNGRDPEGSLSRQQQAGEQFLNIQES
DIPESVDRNTSGNKSVSNASLSQRAPLPLPQNAAVHQFQDAPVPQSQNPP
SPQIENAPLPLSQKAPPPFSQSKPPNGSSSSPGTNHLTSLGDVFVLEINN
VIDSFFLVAEFLPDHHGSNDTKFIQYNSEYRKIYKTSKALHFISLSILSV
MVLETAVKLFCTGCGFFKKKFEVFDAFIVVSSFALDFVFLDSRWYETGKD
ATTILVLLLPWRVVRIVNSFLMTMKHKHHLQMMNMKRAKKKAELKSAKLQ
TLLSEVRKDVQLLVALCRSHDIEEKDVQACLYGKGRRSVTLSAMSTCTSL
MLISTLGKDAIQEDDIYGKVFKEALNEGDNAEIDTEIKQAEEAAIDAAIE
LDEQIEARKKSRKYKSKKALVKRSYTVPRRSASVEIPDPDINENNSNIFY
VNDGYLTAHASSPRNSELLMVESGGVAGVRSGLRRGNFTLVGTPPPLPPS
GSSEENHHRLSCSRVEEESSSTDENSEDALSLTAVTTDLSPQQEQQQIQ>

Aplysia californica uncharacterized LOC101854637 (LOC101854637), mRNA. XM_013082371

MTMEDDSTKNSRSCECTSLQHSVCLESMVITLTIVSALAVTGEMLLDFHF
FTVTETISQDNQSLAGEAPFREDKDVMDILDTVFHYTSLCIAGLFGLEIL
LKIAFLRMRFLRHPWQILDIFVVSGTLGVEIAFHFLDLPYDSLYAVSYVV
LLRLWRVPFVCNIRANLIREELEEDMELYRCGRQKAEERCSWLEENLNQQ
ANIIKGLEQTLLGLKPSSMEDEESSHAETQDNSQPSAAGSHVTTNYGQNG
PMMSEQPSVQSSRKDQNQKKRLHRSKKRVESDNAEMEMSEFNKDNPRPDH
ARKRENIESSNFSESGTKRELKDKGDNSKAEHIDTSEVQLRPRGYSDNML
DFSNTNSLPRSEASLNSSSSTPRPGQINMRQRKRRSSTSEYIDYSNLSSP
EQDKVFYDDVKDKFSSCPSLDHSTQSGEKERSVKSPELWTGKKKEVIEGG
TDEVDGGTKEDADNGSIKKTDSSTSDASSGVSSEVSVNGKRERRDRKKYK
RFTSCPEYVITQRMSITNDESKLLDESHTTEDPSMYDNMAFLNDEDTGLH
VLAEFDGSKTYRNEDGIPMTSL-

Crassostrea gigas XM_011420311 XM_011429833 XM_011440664 XM_011458201 XM_011454844 XM_020070532 XM_011450860 XM_011440929 XM_011436069
Mizuhopecten yessoensis XM_021517539 XM_021506621 XM_021506642 XM_021492263 XM_021492266 XM_021497401 XM_021497397 XM_021497398 XM_021512362 XM_021497391 XM_021497611
Lottia gigantea XM_009053636 XM_009064134 XM_009063093 XM_009055697 XM_009059372
Octopus bimaculoides XM_014933788 XM_014930220 XM_014931773 XM_014930385

Limacina antarctica TR73269_c0_g1_i2 transcribed RNA sequence. GDRM01043900

MVTTHERGGEGSVGGASEDTSHATLKATNSRYRFPHETHNGTKTGDSPTK
KPRRHIGDGGLISILAAQTARNVGSAFTKIEEKIDKEVEDDLLYEKRELK
NKVDRFRHQVEAFTHSKPVLLLVVFLNVIDCIFVGLELVFDFLYFTGGLE
NPTDMKSINGTTCYPETGSFKTFGDLSHYFHYASIGVLSILLIIVFAHVF
GSGKRFFKHKLHTCDFIVIIAAWVLDVVLYKGINSFTRGTAVILLMVMLL
LRVLRVLNSLVVILVDGQRLQIRVMYTTKKKIQAELDDSKAKGGDFKQQL
EHVRTFCLSRGLKEHEFERILNGGAPHTYTNGDLNGNVQKGKGHKENGID
GTPPRHGNVISRQFKKISQVMAPARRSVSEDVSSPTSSNGLSNGLSNGLS-

Opisthokonta – Animalia – Lophotrochozoa - Brachiopoda/Phoronidae
Eukaryota; Metazoa; Lophotrochozoa; Brachiopoda; Linguliformea; Lingulata; Lingulida; Linguloidea; Lingulidae; Lingula

Lingula anatina voltage-gated hydrogen channel 1-like (LOC106176220), transcript variant X2, mRNA. XM_013558499

MDGFKKLHEDLEKVIEKDDSNSSTTTSEMEDPKHSKQTFRQKLRHILHTN
KFQIGVICLVILDCLLVIAELLIDLEVFEIGEAKDELGPAKVLHYMSITI
LSIFLIEIFTKIFAMGLDYFKNKLEVFDGIVVVVSFVLDVVFANQEGAYG
GIGLLIVLRLWRVTRILNGIIMSVKKQSEKRCLRERMLKEAAEQELAKFR
EYCAAQEKEIEELQALLKKHGIDFPKIEKPVEVSTISVTAEVNEVDGYTK
RPDDSNA-

Lingula anatina uncharacterized LOC106178050 (LOC106178050), mRNA. XM_013561049
MADRDDGSRRLEHTIDNVENGTKKQTYQQWLDHYLHCQLANSVILVLVVL
DSLIIYMLLLIDIGIIHALCTCSLAEDNHSSTINGSSTAGFTNKVLPVPN
SQVNLAGEILHYISLCILCLFMLEVILKLVARSGAFFKYKLEVLDAVLVT
LVFVIYVVYSFDIIPSTAKDGVGLLIVFRLWRIREVIRGAALELRKEAIE
KFSRERKARIEAECKAVEAISQQEWDQREISSLKEKLSQYEDRLQPSTQA
PEIKSALKSKSSSTKKTEQGGGRFKKKPTSQPVFTDFHDDHDQHTDDHRI
SFDDKRRLSESKKPKKYQPAITSTAFGYDNESYDGSLDAKEEEKDVDIPL
KXLQERNMGLTYSFNWQHSLFDFVRLLS-

Lingula anatina transmembrane protein C15orf27 homolog (LOC106155662), transcript variant X3, mRNA. XM_013530614
MASLLWRNPSAANCNVKKKKKYQGYICLSSQFSFLRHKSFEQELEDDNID
DDILQTEIEAQAQAEKSCHEKLVEVLESNPIQVAICILVLIDAVLMVSLL
MLDVHIVQAKCNANQEDISKLIDAIDSRMPGALAHVHGADVTLSDIINSL
HGETNSSHGSSSHSVHKRDLSSVAHLLDAVQEGAETVMGSSSSEGGIFLR
RLLYKEETQGDNDSTLEVHVSSDKLAANESHSKDAHGDGHGGHSLIENVA
HALHISSIVILGIFLVEVILKCYALRLSYFRKKMELVDGIIIIISFTVDV
IFYDGLGGRSGVDAASLLIFFLMWRMLRVFNGFLVTSRKRLKFRLTLQMR
ARKRAEAKISDLDVRIGFMEKELDSLRSLASKYGAKNHEVLNCKPKASVH
KNVTAKEGISSMLCASMSMMHHFASKENVAKNEQMAQITESEEVIEDDNS
NRINPFMLSPLKGKASQMNTKDSKENETTVTGSVKPDPVDTNISSPSGQT
KEASALNDVKIDMEDHRENDADIVANPNGKNECSATDDKISSPAPPQPSP
QAQKSKTQDLNERNGTLPSQQSTRSRGALKRTSSVDDQNMSSCDDQGTLP
TSGEPTPAHPPGEVDGSENLQQKSDTQTPSDCNKLNNVAAKNATILTNGD
VISNGM-

Lingula anatina uncharacterized LOC106156217(LOC106156217), mRNA. XM_013531343

METQIISRQDSVAMEKDKTWEKAAKVSFRKRLTKYLYSYPLLMAISILSI
ADAACVVGEVLIDLTLTNGKTEAAESYVTSIRQALYDRFPHLKSIATESV
SDLIRKISNIHCPRPTTSESTVSHAPTQTEEPGVLYLNVLTGSLTKQAVP
GQPSNCSLPGDTDQCPHALEHVLKEIGHVLHMLSLFILSSIVFVHCLRII
ATKRRFFQYKFQVFDAAVVTISLVLDLAFLKGIWSDDTGEAAVLVLVLVP
WRVIRIVNSFVMTVKEKDHIAMKMVKSGRKKALKRVSDLIKQAERHKQEI
RALRGLCKKFEAPEDAINACKPQVGNENRFRRRSSGASLTMLASLAAFGS
LGLDPSKVHPSDDEESEDGSQPYLPTSLHEKSSSVNSETTENRAVDDDSP
SNNNFGDSFFSNDDTDKNALDFYNENETCVELQSTRQEQQKENDKFALVE
LPEKKPRSFSVSSIGTASSFLKSPIKRLRSLTYTEGNGADSEPRSSHTAT
LVDNMEIPNEQNEITHC-

Phoronis australis GFSC01012821 GFSC01079495 GFSC01010288 GFSC01051093
Phoronis muelleri GKAW01058824

Opisthokonta - Animalia - Lophotrochozoa - Annelida
Eukaryota; Metazoa; Spiralia; Lophotrochozoa; Annelida; Clitellata; Oligochaeta; Crassiclitellata; Lumbricina; Lumbricidae; Lumbricinae; Eisenia.

Eisenia fetida Ef_Cf_16012018_18296_c2_g1_i1, transcribed RNA sequence. GIKG01069578

MVQMRMEGFKKVKPSDDMERVIEKDDSNSSMTTEYDEGKAYPAAWRDHLS
LTLDSIKFQIAVVCLVVLSGLVVVAELLISLNVLELHMQSIVPQVLRYIV
IGILSVFVVEVVLKIFAWRLSYFRNKMELFDGAVVAVTFALSMPFSSNSS
FHSSIGLLVLLRLWRIVKILNGIILSVKAQAERKLRHERHIREALEQELA
KFREYCASQEKEIELLQAVLRKHEINYYPAEKPVAVETISVVAEVNSICE
DQQQQQQHEFHCEFAGR-

Eisenia fetida 59034, transcribed RNA sequence.
ACCESSION GIUK01058977

MVFSMHRVSQYGGRCDWLKNDQFLRDLEGDSVDAEILDEELRLLQEQQPK
TCRGKTARVLETSPLQIAMCVLVLIDAGVVIAEILLDLHAMRSQQRAATS
DLVQAMVFLKDQYSADLGDYHGEGIGYILDKIRLKQNPANGNSSTTAETN
VTRSKRHQTLPQPDKWTLLLATSVPVMPTPFEVGLREKPSEPEPLESLYG
SSKVHKGPSFLDTKVPSFMPGHSRADVDPLSVGLARVSRRAVNGFSGLFE
PRRKRYIRQARHKNDQDNDFVIARVTRTPVVGSRDEGSSSSSRGFIYTEP
DSDAAAVKRSAELASESVVVRSHDNEEDDDYEGKADGGDHHLTESFTIKN
TYAEKLLKIAHILHYGSIAILGIFVIQVFLKIFGMGPEFFKNKLEVFDGV
VVMASFTIDLIFVEGITGTEGEEAIALIIIFLLWRILRVINGIMVTAKKR
QEFRIKLQKRACRRAEKKLECLDDELSWKEKEIHNLKNLCLRKGASEEDV
VRCRPKRSPFFAKMDTSAGLSSIASLSLGFTMSMRSSDSRMRLPIAHSLV
SLSGHRQTVHDVHSSSLGNPSMSLNRSGQWPRSRKPSTSTVRSSDNQSLP
VSMRQLTESRSMEKIAEERPKHQLDLSIMSSSTSMQSLAADSISNCCPVT
SGLGLLPSRSIANDRVVAAAVSGLTACNSSRPQPSESGGGIPDICIVISD
ANGSTQVNSSRERKRSACQAASSSDDSEGRPSPAESVLSNPDHGNNRTVQ
FYVETPTDMSMENIASTSASTAGAANGDGRCANDFSSPSTSSQIVNGPAS
RLPSPPADTDRQQQQQQRESGGSLTSRWLSKLRRSSKSRSHHENQVASSS
DKVRRKPVWVSPFAQAKEKKTKETFL-

Glycera dibranchiata GASB01008782
Megasyllis nipponica ICSJ01060984 ICSJ01016660
Platynereis dumerilii HALR01262921
Notospermus geniculatus GFRY01018744
Alitta virens GINI01122785
Lumbricus rubellus GIKI01097867 GIKI01020978
Arenicola marina GJHO01023177
Paraescarpia echinospica GHDM01078138
Hirudo verbana GGIQ01040411 GGIQ01076257
Harmothoe impar OX381720

Opisthokonta - Animalia - Platyhelminthes
Eukaryota; Metazoa; Platyhelminthes; Trematoda; Digenea; Strigeidida; Schistosomatoidea; Schistosomatidae; Schistosoma.

Schistosoma haematobium Voltage-gated hydrogen channel 1 mRNA.
XM_012937229

MDESNKELINNVPHSRAKTRPSGKCTLARKRLKQVFNMRKYYLSVIGLT
GFEALLVLCRVILETESLRLPPGNTQRLILEGQLALECLSLFTLTLFVV
EVPFKIWAMGIRQWGRQLLFIIDGLVCAVCFSLDIYNIYRHSSRPSRGI
TSSKVLELCNYLHITSQADTASTFAEIFGLVIVYRLWYIKRFIKKTVFI
KSKQSIKRIQELQQVYGEADQRINQLENILQEQIDRKDNHRSISNVILD
NKQGQQQTTRPMSKVNYSNRPAPRQFSNIER-

Schistosoma haematobium hypothetical protein mRNA.
XM_012937230
MSMIENDERNFSTKKENTMKHKRRQISTESNQCQSDVTNGLLLPVNEII
EIPIETILTPAPPPTPPPPPTTTTTTTTPTVTINTIQSCNEKYKLLFSR
ILDCKLFHMIIVGLCALDGILVICMLLLEIESLKLKLTHLRYRLNFTSF
IFECISYTIILLFLIEIPIKLWTFGYQFYQYQWIELLDVFVCIISFTVD
TYNIHRHIMETKLNKMNNTMNEYTIDDNLEQTLHTTIADAAGLLVLFRL
WRVIRIVNSIIVSVTATHERNMKSLKEAHHISLKRIYELEQLLQDNGIS
IPSLTPKSQSILAKIF-

Schistosoma japonicum FN318210
Schistosoma mansoni XM_018790876
Opisthorchis viverrini XM_009166151
Heterobilharzia americana OX104105
Dicrocoelium dendriticum OX104059
Echinococcus granulosus XM_024492501
Spirometra erinaceieuropaei LN077116
Hymenolepis microstoma LR215995
Macrostomum poznaniense GIJT01038552

Opisthokonta – Animalia – Lophotrochozoa – Bryozoa

Dendrobeania fruticosa dbe_tr88007_c2_g3_i4, transcribed RNA sequence. GJXY01266092
Eukaryota; Metazoa; Spiralia; Lophotrochozoa; Bryozoa; Gymnolaemata; Cheilostomatida; Flustrina; Buguloidea; Bugulidae; Dendrobeania.

MKGIGSFGKAGPDDLERVVEKSDTSSSIVSDDIVKPPQTIREQIAEIIHS
RKFQIVVICLVAIDCLLVVSELLIDLKAFEQEAKVGHDITKSTSSGGEHV
TNGEGSNVKKLEDAEKREKASLLAAEVLHYFSIAILSIFLLEICAKLFAM
GRQFFQHKMEIVDAVIVIVSFALDLAFIDHERLASAFGLLVILRLWRLGR
IVNGVVLSVKTQAEKKIAKEKGLREATEAELVKFREYCTAQEREIEALQL
LLTQNKIEFTKMAKPSLPVSTIDVVAEVNQFIEATKLASGGGGEGGGDSS
VL-

Dendrobeania fruticosa dbe_tr82209_c1_g1_i3, transcribed RNA sequence. GJXY01243660

MTGSELMEIDEKLSRALREDSVDPAVVLEELKSLNDDVLDHSNTRCRVRL
ERVLESTPLEIFLVVLVIVDVIILLAMLLMDLNVLHLYLEDGNNAASKLS
TALQTNCRGNPELNLYNKSNLIIITRHLEHDGCIWDPHSTNYTISDSASS
EAQSPNHVTQHHRRRKRSAAPPKEGSNPTIAILLEAGHILHITSVLILAI
MVVEVVLKIFALGAKYFKGKLEVIDGIVIIISFAMDLYFIDGIPSEGVNN
GATVLIIFLLWRILRVFNALLVTAKKRLQFRIRVQKRMRKSLEEKIELLN
DDITHYEDYIENLKRLAKRHNVPDYEMKACKPLVRQLKKANTSAGLASMM
QMSMGLMQGMNNMKVADKPRSRIVDNLRNPPTETVETDKESASAADVLVN
AMDATMAANSTVDVVHDTSQHILPVPMMTAMATSNGSVHRSPHRSPHRSP
RLPHTSTSVANGTRTIPSMEFAKAVATGSPKSNEALLLNWNDKSDKENND
PNSNQNITSLS-

Dendrobeania fruticosa dbe_tr59641_c0_g2_i1, transcribed RNA sequence. GJXY01200275

MTIPHDPVQRKPPAKKQKFHESLAKICVNHKFMLFVLIVSVGDALLVATE
LTVDILAIKIQKEEVCDTKEILKYLKREHSGQLDELFFNNSIKAILYELK
TRNDHHYHHKRDVSAVLESEDIKVRRKRAAPPKKGKGEIKESPLSSVLLF
EVAHACRYGSIALLSCMFLINIFRVYAMRKDFFHSKLQVLDAIVVIISLL
LDISFLPQVWTFTDATLTAIPIIIGLSWRVVRVINNSLINLHEKDKILLS
RETALLKDSEKRNRHKKHEIYNLRGLCRKLGAEETDITACAKLVKTIKKK
RFSKISLASGIMSVNSLAFLGTLSREKQPHEPNLDRVESFTKTDETKSDE
GFDTLQRSSEFLRRNQRGSSSNKIPARRAGSRMSNVSSIGSISMASSFDR
DLDDDIDNTSYERATETSDTDNDDSDGRGSKSDKSDKSQRKFF-

Terminoflustra membranaceotruncata GIMX01037542 GIMX01141019 GIMX01229943
Fredericella sultana GHLZ01039460 GHLZ01006033
Membranipora membranacea OU612068
Bugulina stolonifera OW285188

Opisthokonta – Animalia – Lophotrochozoa – Entoprocta
Eukaryota; Metazoa; Spiralia; Lophotrochozoa; Entoprocta;
Loxosomatidae; Loxomitra.

Loxomitra sp. KK-2020 isolate Shimoda Marine Station
tr115673_c0_g1_i2, transcribed RNA sequence. GIMU01079853

<VEKQLEEAEADASRQEEEIASGLRRKIHDLIHKRWFQIVVVTLVILDSL
IVVAELLIELNVLGDVMKKVCNGTGHEIEELNEEFLFAAEVLHFCSIGIL
SIFLVDLALKLYAMRLDILKHKMEVFDAFVVVVSFSLDVAFIGMEGGEAT
SLLIVLRLWRVTRIINGLSITMKSQADKKVRRLIKIKEALSLEVKQLRVD
LARSELLIKRMNKSLEQNGLEVPGVNENSVLRKSLLPVRTELYTDCSDV-

Loxomitra sp. KK-2020 isolate Shimoda Marine Station
tr112662_c0_g1_i1, transcribed RNA sequence. GIMU01076890

<VILFLFIIELLLKLFVMRWEFFHHKMEVFDAVIVIVSFSLDIAFINQEV
AQASSLLIILRLWRVTRIINGFSLTIKTKSDREIHRLTRIKDFLHTEVKR
LRMELARVESQNRKMIRVLRRHEIQTPILPTSEVISPK-

Loxomitra sp. KK-2020 isolate Shimoda Marine Station
tr203811_c0_g1_i1, transcribed RNA sequence. GIMU01203676

<AFEGINLLMILRLWRITRIVNGLIVSVKAETEEKYHHILHHKQAVIDSL
KSKLDRCQSLMVKHSVTIPEGLLHSMDELDAPPFPAITPSTGNVCATCNI-

Pedicellina cernua GIMK01062983 GIMK01039117
Loxosomella nordgaardi GIMJ01039792
Barentsia gracilis GIMW01064503 GIMW01098986 GIMW01061213

Opistthokonta – Animalia – Lophotrochozoa - Nemertea
Eukaryota; Metazoa; Spiralia; Lophotrochozoa; Nemertea; Pilidiophora; Heteronemertea; Lineidae; Notospermus.

Notospermus geniculatus TRINITY_DN199650_c0_g2_i7 transcribed RNA sequence. GFRY01018746

MDGFKTFKKLDDDLQRVIEKEETSSSVTSESDETKHAMMDHREHLKHLLH
TNKFQICVIILVILDCVIVIAELLIDLKVFEMEGGKNAHDSIAPHILHYI
SIAILSIFLVELGVKLYAFRLEFFKSKMEVFDAFVVVLSFALDIAFANDE
GIIGGLGLFIVLRLWRVTRILNGIVLSVKIQAEKKLARERSSREAVEQEL
AKFREYCSAQEREIEILQGLLKKHGIEFQKMERPIVINKIDVVAEVNEYI
EKTNSENFSSA-

Notospermus geniculatus TRINITY_DN203828_c2_g3_i1 transcribed RNA sequence. GFRY01079887

MKTSSSSGTVPISEEDTTESPVSPSPRLVRHESLDTGTPKQNGSVASSLH
VKFTVGASDDEDDEPHYRRPRSRKNSSFGILDLNEDWITRNHFLEKLEAD
SLQSDVIQAKLAEIAEDTVKEKETCRRKLAKHMTTTAFTIFILVIALLDA
TVTITAVILQIHSEHDEIIVRREQHKKFSKFTKLAYGNKVDLDNVLSGKE
MIDRVEKYLNKTQNATASAANATKNIHKRDVSEEQQYYKTIEISRRGHGS
RQGRSAKGQGRRKRRSETADDKDRGERVLVRRKRAAATAADGTPLFESKT
ALTLANVAHNLHYCSIALLSILLIEVFVKIFALGCEFFKSKVEVIDMIIV
SVAFFLDVYFIDGVMDNVAATILLFIWQMIRVCTTLILQDRKRVDFLLDV
VRDSNDANKKKVQEYQERNNSLETAVQRLTAVAKYNGATEAEIKRCVDII
VTVNKVEETKKSPKKPMFKSLQSVFTNVKKRRKKKAGFDLDQTESTDSKS
NPPKRRLSTDLNVQLVHRQRMASHANIHPSKTHRHSLHILHSLHHHRHDR
RRLHDTDDIKQNGDYHPEHGSGEGSFRSPVNRRRLHLLGGHTNLEHEEDL
TSPRFTFVSSFSPAQSPSVSRTNSILKDCSSPRFKKKDVCSPNVSRSSSL
SRSQNDSGFDNCGFSKADEHQERGKPSDIKGSVNGDVGIGDVRGNLPMNK
EGDTGVSSDGTGGKEGDSNDVTFKNSGDTKSERECERSRFSDSVTEALSP
QSNTNHMKTGDSCQKKLHGILKQPSFEVQPADNQNKPSDEAHSPTVDTAI
INDDDEDTGL-

Notospermus geniculatus TRINITY_DN198369_c0_g1_i2 transcribed RNA sequence. GFRY01070393

MELMKIADNINNRNARKPRLHPIRKEPERLTKIESLRRRINRFLNSHHVL
ILICALVVIDAAMSIGQLMIDLYMIREKFGKAEESLLHLVDILKKDYPYI
NAYQVNTIEDLLHAIDYGKRDLKQTPEKRTTRDAHVNLNEAPPNELSSSV
SGLPQQMWNYKRKREISPRSKDKRERRDDVTNVPFFQTSAEGKSQSLRNI
LQTSSESQATGFHASVTMAVDDSLNHRTTSLPYTAVVINDKTKLPPKGGA
MKEIGGKSVDLDDPVIGYTVNKTIASDYMRSKTGNSSCDCSVVQEKNSTQ
LCHCEDDYHLLMEIAHSFHLGSLCILSLMTLEKFMKGLSQGKSFLKRKLE
IFDTFVVISSFVLDIVFLQGIWSTAEKDAALFLVCLLPWRVIRIVNCFVM
TMKQRHYIRMQLQKHARRKAQKKVKLFKKHLERLKRQIKSLKGLCRKHGA
EEREINACAFVFTESRRRKSSLPVNAMSTMASLALIGVIGNDPHMPRQQD
DMEEVDLDTLDEKDEEEIEELDEVENELMRIEEEPVDQGVQMNGNCHRDR
KVSTISGTESIEISCGSVSFDDSGSPTMERRLSDDSIVDIGAISDDSYAD
TGDSDGGNRKDEKILKSRRFSTTDFLYNSKKSFKKRKKKK-

Opisthokonta – Animalia - Placozoa

Trichoplax adhaerens hypothetical protein (TRIADDRAFT_54341), partial mRNA. XM_002110878
Eukaryota; Metazoa; Placozoa; Trichoplacidae; Trichoplax.

MIDDGDNNSETLFAVHGDNEGMNPPQSVTWRSDHRAKLRQLIYSHKVHIA
IVVLVILDALIVIAELLIDLSVIKVHHTSPLARAFHFTSIAILAIFLVEI
VLKLYASDLAFFLHYFEVFDALIVIVSFVLDIAYSNSEALSGVGLLVVLR
LWRIARIVNGIISSVKSQANDKIHHLRRELEKTRSYLEQKLDRKEAEIQL
LKKVLTDRNIPIPKPLLEVDLNGNSTAQANGSASEEITVDIAGSSAKSTS
SAYIG-

Trichoplax adhaerens hypothetical protein (TRIADDRAFT_54343), partial mRNA. XM_002110360

MSKKVKTEDKDSYIDILDEESASVRIDHANHTLQQNSDCLHKTEEIMEHP
ITQLILILLVFIDCGIVIAELLIDRDAIRVEDVEDVKEAFHYTSIMILGI
FLFEVALKICVEGLAFFLHYIEVLDALVVIISFIVDIMSLVPYFYNHVFN
LICYARAAENKLRGIGLLIILRLWRIARIVNGIIASVKKHMRKRLETITE
DRNRCRRKLNRAIKVARAKVEEIKLLEGILEKEGIEYRQIVAKTEQGDGD
PAEVKLVIEEDIIDDKDNLEEEHKSIDGKEESKSNQEEKSKSIDEKEEGK
GNQNEEGKSVDHKEEDKSLETGQTEKSEKDAQEGDKRSSRGDSDAASHHS
ERKADDAKDNKEEVEPEVLVYT-

Opisthokonta – Animalia – Cnidaria

Actinia tenebrosa voltage-gated hydrogen channel 1-like (LOC116299624), mRNA. XM_031708302
Eukaryota; Metazoa; Cnidaria; Anthozoa; Hexacorallia; Actiniaria; Actiniidae; Actinia.

MDPDDQQLVGRLSFDELSTDTAEMEVGGAGDSNLEVVPSTPWWKDNRAKL
RELLHSQKAQYTVVGLVVLDCIIVIAELIVDLQILKVHHDNPAPHILHYI
SIAILSIFLIELILKMYAMGLDFLRHKMEVFDGIVVVVSFALDIAFSGSE
SAAEGASLLVILRLWRVTRIVNGIVMSVKIQAEKKIEQLTAENDELKEEI
IKIKTRNAELEKEISALKGQ-

Nematostella vectensis XR_007308022
Acropora millepora MZ029046
Pocillopora damicornis XM_027201316
Stylophora pistillata XM_022939457
Dendronephthya gigantea XM_028555994
Xenia sp. XM_047006720
Hydra vulgaris XM_047284425
Acropora digitifera XM_015907824

Opisthokonta – Echinodermata

Anneissia japonica voltage-gated hydrogen channel 1-like (LOC117105210), transcript variant X1, mRNA. XM_033246280
Eukaryota; Metazoa; Echinodermata; Pelmatozoa; Crinoidea; Articulata; Comatulida; Comatulidae; Comatulinae; Anneissia.

MDTKIDSTNDDTPFQETKKSGEVKGTDMGFLNLTGNSKSEDQENIVKNEN
IEVRTAPPNQPAVTESADTFRGKLQRTLHSHWFHGAIIALVLTDCILVIC
ELVLDLSAVENENKACEGEGDEHKEEDTAEKELTAALVLHYMSIAILSIF
MVEIVFKLYAFRLEFFKHKLEVFDAVIVIVSFVLDIVFLIYEETFMAVIQ
LLIFLRLWRIVRIVNGLVISVESKAHEKITAQKQLREEAEEELEQLRKYC
DQQERQLELIMAEARKHGIVLNSISKLDPPKHRKQFKVDVDVNGPPLDST
TNKKSHQDGFTNVTFDIQDEPADGKTGIS-

Acanthaster planci XM_022240659
Strongylocentrotus purpuratus XM_030990962
Patiria miniata XM_038200083
Lytechinus variegatus XM_041600440
Asterias rubens XM_033772565
Paracentrotus lividus GCZS01131405
Echinarachnius parma GAVF01025219
Evechinus chloroticus GAPB01008884
Holothuria scabra GHHS01281259
Loxechinus albus GGVM01158715

Opisthokonta – Animalia – Porifera
Eukaryota; Metazoa; Porifera; Calcarea; Calcaronea; Leucosolenida; Sycettidae; Sycon.

Sycon ciliatum, transcript: scig1.0025458_3, transcribed RNA sequence. HBWS01076755

MDTDYQSYKKYEAKEKERNEEEDRVAENINLSIRSWRDLIDREHLKKFLT
YSQKVQIFIVVLVVLDCTIVLTELLIDLGIIVKNLCEHDSDCPLLSISPN
GTINTTETEGYYKCFKNLEQTKCEFHDGKKRCLSEDISPDIDPAYVLHIF
GIVVLALFLVEIILKLYALGLEFFHHKLEVFDAFIVIISFSLDVAVSGNE
DAWEGVELLILLRLWRVARIANGVIISVKKEAEKKAEHLQGKVEAFQEEV
RKLKEKLAEAKLKGYTAYEDSDNEATPSSGGESQAQDLHVPYNAPGFVPA
TDTAEA-

Sycon ciliatum, transcript: scig1.0041756_2, transcribed RNA sequence. HBWS01116653

MADSDKEGGGAAANDSSITLETLHQQDSVGSPPSDTTTAASSASSHGRGV
DIKISVDDIDGPRENERPPSIEENTDAHHSDSNPGSALVPPNSADIKRVA
SVEIVPPEELPDIDDENFDELQHVYDDETVVQLKDAWRSRKHLANFLTLN
HYFHIFVIVMVALDVMVVLVELILDLELIRDNECRKDSDCPITRRSNLSD
TSSIQSCSYDTNFSHCIYIHGKHSCQLEETHHDIDPAEVFHYLGIAILSY
FNLEVLVKLFALDIKFFKHKLEVFDAIVVITSLVLDITIGGEGARESVSL
LILLRLWRITRIFNGITLAVEEREKEKRHKLKHDKDLALKDVDRLQKIVH
ELKVEIHEWRSRAHVEPTYKGLRHADELQSMIAEEEKKHHADGKHHGHHH
HHHHHHGKNHHGKNHHHGHKNHLHGNHMQAPSSDSNHSLNSHGSAQSADK
AV-

Sycon ciliatum, transcript: scig1.0080075_3, transcribed RNA sequence. HBWS01098561

MSDVSAATEAGTPGLFEVLSDAEGARSSSGNTSRQEDTAKSASPTKSETS
SSGSATKSGTSSRGDPPAYDDDHHAAYIAQQKAAVHRTTFELDGRPLPDL
EHHYYDEFHDPEPEDEPSHDFKDFWRSHENFKAYLTKNHHFHEFIAFLIV
LDILIVLGELILDLEILRADECAHDHDCPLVEATVNGSMWDCSFNSNYTT
CLYKGGRHTCVDEYTKADKDPSEILHKLSIAVLSCFVLESIVRLVVLQRE
FFKRKLEVFDSIVVTTALFLDIFVHHGAAVALLLFVRLWRVTRIINGIAI
AMESREEAKRHELRKQNRCARHELVRKRETVHLLRIELMEWKKAAAVFHE
AEHFTVAAPMPTG-

Oscarella lobularis GIUN01026972
Hymeraphia stellifera GKDX01077406
Cymbastela stipitata GHWA01021218
Aplysina aerophoba HANI01325610
Leucetta chagosensis GIYV01003700 GIYV01008849
Eurypon sp. GKDW01052485
Dysidea avara HANJ01327516
Halichondria panicea HBWD01528479
Halisarca caerulea GFTQ01354451

Opisthokonta - Fungi
Eukaryota; Fungi; Dikarya; Ascomycota; Pezizomycotina; Eurotiomycetes; Eurotiomycetidae; Eurotiales; Trichocomaceae; Talaromyces; Talaromyces sect. Bacilispori.

Talaromyces proteolyticus uncharacterized protein (BGW36DRAFT_366576), mRNA. XM_046214761

MASSADPSQPLLGDQQQIERRQQQRSDDVTRKSSSPSASHDLLRRITES
HFIGNRMPSWSSRGDGGEYGHRYHDYYNEYYREEPSSSIPRLRAKARSAL
SSKWGHYAVLFLVAVDVACIFAEFLIELHTCELRERHQVVDRRWEIAQEA
LGLSGLIFSCLFMVELIVTVLSFGLHYFKSKFHIFDAIVIVVAFVFDVAL
RGLVEELGSLVVILRLWRVFKIIEEMSEVSAEMMEKYEDEIDKLKHENMK
LKRKLRGYGDEDDDMEANAGGDEEEDNND-

Aspergillus ibericus XM_025718342
Botrytis fragariae XM_037341616
Aplosporella prunicola XM_033538120
Grosmannia clavigera XM_014317838
Hyaloscypha bicolor XM_024883444
Phaeoacremonium minimum XM_007913592
Penicilliopsis zonata XM_022728519
Lachnellula hyalina XM_031151652
Venustampulla echinocandica XM_032013960
Sporothrix schenckii XM_016736437

Opisthokonta - Choanoflagellates
Eukaryota; Choanoflagellata; Craspedida; Salpingoecidae; Salpingoeca.

Salpingoeca urceolata comp17136_c0_seq4 transcribed RNA sequence. GGOY01009290

MATKADDPFSASASIGRSTLTLAMSALGVSNAAEVYIRTFPIGAVPRVLS
GPAKSFRQRVHDWLERKHVHNFLLFLIALDVLIVLAELLLEFEDCRLELH
DLEHDSHSNEGKIERIKKAEKGLRIVTLIILSIFMIEVAAKVYVLRQTLF
RHKLELFDVIVVIVSFCIEVFLKGFERRAASIVVIFRIWRLTRIINGVAV
ALELRMEEKVTMLKHAVFDFRQKFSVAEAHLASLRAQLANVQAQAASHLQ
DEVAEMQSTTRRVQRLLTEPVVSEELETVLSTSGRFEKAQEGYVPPGNSL
LVSGHGPDPLAPYRAKSSPGPTNTLNKSRVTWQVTDSDDENGDAEHVYRA
RSLPTHDLPTPQDSVELHADTNTQDDPESPQPGQSPDVGWASKV-

Salpingoeca dolichothecata GGOK01012774
Salpingoeca kvevrii GGOX01017848
Salpingoeca punica GGOZ01029229
Didymoeca costata GGOQ01021484
Stephanoeca diplocostata GGOS01015804

Opisthokonta - Ichtyosporea
Eukaryota; Ichthyosporea; Ichthyophonida; Amoebidiaceae; Amoebidium.

Amoebidium parasiticum JAP-7-2 G13375_050632 transcribed RNA sequence. GAKF01050632

MVYGNHLQRHQTRVAGGFHITPHLVLKEPQFEKAEGKHWRNQLRDFIHSK
RVHTIMLVLLVLDVMLVITGIALEIQNLSGALHLCESTVESCVAKESCTM
RDFENHALVTAEHWTAYLSLAILAIFIIENLLLIVALGPIGYFSNLLYAL
DFVVVITSFVLETLFIDKPEEGLLVLYRVWRFVRIAHTFYETNVDEELER
VKHAIKQAERELLAMSLTPEAQKEAHEALAKRMAESYPDAAGAILSAALH
YAEHNKHGKTPQSDPMEVKSSTEGEEEATVQQRTP-

Archaeplastida – Chloroplastida/Chlorophyta/Charophyta
Eukaryota; Viridiplantae; Chlorophyta; core chlorophytes; Chlorodendrophyceae; Chlorodendrales; Chlorodendraceae; Tetraselmis.

Tetraselmis striata, TRINITY-DN8512-c0-g1-i1, transcribed RNA sequence. HBPS01066517

MERSGSSAKAIKPVRLDRKSMTVLDRQLSIALKREQSTKRLQQQQSGGRP
AKEQPEGKGAVEGAEEEEVKHHWRRRLLHFLHIRWVHTTMTALLILDLIT
VVTSLELQIQSSQKKGQAKSICLHEYETGHAMPTYMENNTTCNPDGLHAC
DYTEQEPYHMAHTLHSAELGLAYISIAILSTFLLENLAMVAALGWNFFRH
FFFVLDIVVVAVSLALEIVAVASAHLELTVGNILIVARMWRFFRVAHGIY
FLEHSEEAAHEVDDKDAGDSMGKKQSQVENGTTV-

Ulva lactuca GFUR01058327
Ettlia oleoabundans GFXW01038773
Chlorella sp. GGGA01109849
Nephroselmis pyriformis HBHO01063905
Chara braunii GGXX01073780
Chlorella sorokiniana GAPD01037882

Archaeplastida – Chloroplastida/Embryophyta
Eukaryota; Viridiplantae; Streptophyta; Embryophyta; Bryophyta; Bryophytina; Bryopsida; Funariidae; Funariales; Funariaceae; Physcomitrium.

Physcomitrella patens voltage-gated hydrogen channel 1-like (LOC112276685), mRNA. XM_024508236

MDAHGRTDLEGADWDDSATTALLPEREPQDAGDGEMPDPGDASSLGSALQ
EVSKAWRKRTQAQRLSAQRWIEQETHVPRRARWREELAEALEATWTHVAI
VVLLLVDLAATAIDILKTMHNKSHDLDVCVDLVESCQGCIGHFEHSAEWK
WTYWTSIVILVILMLNVLGLIVAFGRSFFLHPLYVLDLVVVSTALGLEVL
LDADTAGLIIILTLWRIVRVAHGIFEVTDEAWEKSIRELETQVKGVQDAY
ERAQEALQEKNRELGEKDGRIAELEARLESGSTPF-

Physcomitrella patens uncharacterized LOC112285145 (LOC112285145), mRNA. XM_024525718

<LNDCIAMVESCTSCGSYFERREEWKWTYWTSVAILSLLLLNVFGLIVAF
GMAFFLHPLYVLDLIVVCTAPVLELTLHTDTAGVIIMLTLWRIVRVAHGI
FEVTDEAWEKDIHRLETQVQAVQSACDEEQVLLQERDQQIAELEARLREL
TVIET-

voltage-gated hydrogen channel-like protein [Arabidopsis thaliana]. NP_001321473

MNIINTGTVDNVEFSIQNLIKSWCRRRKWRQLCNFSPKQQQEELISINQQ
WRITLSNFLESYQVHLFTIFLLSLDIILTSLELSSSLLSCTSVKKTETEN
EWFRWGGTVILSILAVKSMALVVAMGKSFFKQPGCVMDGTLAIVALILQV
LLEKKGTGFIVVVSLWRVLRVVETAFELSDEAIEVQIDGIISQFQALSKE
NRTLLETLAEKDEVIKMLEEELNRFKENGDIPFVKP-

Selaginella bryopteris GEMU01010353
Bryum argenteum GCZP01005159
Sphagnum palustre ICRE01047567
Triticum polonicum GEDT01074033
Ginkgo biloba GHLL01415143
Marchantia polymorpha GEFO01020457
Selaginella sellowii GIMF01015262
Dicranum scoparium HANF01033133
Isoetes echinospora GGKY01088505
Lygodium japonicum FX959670
Pinus sylvestris GHKW01007963

Archaeplastida – Rhodophyta
Eukaryota; Rhodophyta; Florideophyceae; Rhodymeniophycidae; Gracilariales; Gracilariaceae; Gracilaria.

Gracilaria vermiculophylla Cluster-14487.30734, transcribed RNA sequence. GILD01028259

MATDQQEAAAAAKIGCAPLKQANLQQPQPMRRPSLASSSRRGSLLSTNSM
YFGIPTTADAEDHAEERHGTHSWRYRVVAFLHKPRVQKIVMGLLLTDVIL
LFVELFLLATFPHCSIIERDGLSCVPANGTTIAAAAAAAAAAEQEEVEHR
WLLVAATRFLAGGGGSGDDHHGDSAICEEGYEWNGLEAGCDEYKWSVVHT
LETFIFSLTVIILSLFFIELTVEMLALTPQIFFRQFWFLLDYVIISISLA
LEIAFRVKREDVYQSFAGLLVVVRIWRFVRIGHGIVEVTHENEENGMREL
FQYVQQLEGLLQTNDIALPGCAQHIHHVHHDHAQHLLDVIEGSVAKQTPS
RVRFAGEDSDIIMDEEGKGDDESSAADPEKRWDTTSL-

Rhodella violacea HBJD01001165
Timspurckia oligopyrenoides HBFP01003029
Madagascaria erythrocladioides HBNE01039390
Devaleraea ramentacea GFTF01008765
Devaleraea mollis GFTE01010127
Laurencia pacifica GFZU01160101
Erythrolobus australicus HBGI01005155
Rubrointrusa membranacea GFTD01034059
Kappaphycus alvarezii GGUP01041343

Sar – Alveolata
Eukaryota; Sar; Alveolata; Dinophyceae; Gymnodiniales; Kareniaceae; Karlodinium.

Karlodinium veneficum voltage-gated proton channel kHv1 mRNA, complete cds. JN255155

MDRILHHAVHTVHTSKSARDAEGHGTWQSKLNEALNSSKVHTILNVLLIC
DLMTVIIGMLLEQYYSDSQVQGLTEAFKDCLEKRTFCPDPSHLAHYGNHD
LHEWAERMEYASLAILLIFLLENMLLVLANGCRFFANPFHILDIVVVVVS
VGFELQGILGEGHDAGIGLVVFAKTWRFIRLGHGIHEMHEEHEAEDHGEH
RVSDAAGSLEAPLQKGSFEQHAKGTSGVHHARSQASSNREGREGCCVQ-

Karlodinium veneficum, TRINITY-DN53119-c1-g1-i1, transcribed RNA sequence. HBOS01067516

MWRTASATGTKKSKIRRIVPHILDEISHEATHFRRLLLHSKPVHFTILVL
LIADGLICLTCGVLEAHYLHGKSDDCQNYVNKCLIHHRRLVNWPGRPELE
LPSWLRSPWMSDAGAEEAWASNAELLLDEEFQDDQAPRMLSGSASSSSAS
STGSSASSSSGSHGSSGSHSSASSSSGSHGSSSGECAGHPHFGDHTLHDI
EIILAYISVGILSLFLVEQVLLIVDLGKEYLKPMFILDFVVIVSSLLIEI
LVVNMTIGGLLVLARTWRFARVGHGVFEAKEMIEEMIGEDDDTVKNMMDA
WKELTPERWEEILHSGSAEILRESGVTPAEIKLGEALGNSPAVAMRALAF
ARGWKQKLDKKKAKRLSGSNPKRQMPGASLQTTATVGKGHSEH-

Alexandrium monilatum, TRINITY-DN21304-c0-g1-i2, transcribed RNA sequence. HBNR01032293

MLAQGRCVHSRVGGQPCLRTLAHARSAGGAMGHEHQSEGEDDILAAGSLI
ALPLRFMNSTAGHLLLFFLLVLDVMIVVASGFLDTQYLLSQTKDCKGYVT
ACTNHSHSRRLGNDGREEQHARLRLLASSGGGSSAPVDCSADPHFGDHGL
HDAEVILAYISIGILGVFLIEQMLRVVGSGCRYLAKPLHVLDICVITLSL
VLEVLVTHLPLAGLLVLGRVWRFARTGYTTAEGLHDIHKVRPAFGSMAHG
AIEAVWPRLPEHRWKALASRSSMELDLEMMEVNLAAEIAKTSPGFVLHAL
ARENERLRTPRSNPAVLTEMTDATEAS-

Karenia mikimotoi isolate RCC1513 Kmik_DN141715_c0_g1_i1, transcribed RNA sequence. GJRC01038612

MSGKVHPEMAVSGESQQVDTVKAEVQEVKEATLDDEATLDDDAYNEREDK
ETNRLKQLVEQMRRENEADRLQAQQRRAALFVKAKDEEKEMGHDHGKHSW
QTRLLHRLHHHYFQGFLICLLLLDVVVVVVELFLEAQYPDCDIIKRDAVS
CVPIACAPSTHGSTSSHSSSSATTSSHSSSTSSGSSSSHGRRIAGMEEGD
WEYGQDAWSWPAEEPLGLLSWESRGRLLSGDSHGPTCKDSHLYSPMESAK
ATCDEHKYGWLHTVHHLLVGVSVFILGVFFVELILLFTCLGCGFFKNPLY
IADLFIVSVSLILEFLLMSFTEQSLVSLMLFARFWRFVRVAHGLITSVHE
PMSHKCEHMEDIVEQLNARNEKLARAEKRHMKMIDFLLKDSKDPKAAKAA
ALKAAKAAMAIEYPQAPDHDRGPRESARAPEGDAAPFLAEHSER-

Scrippsiella hangoei, TRINITY-DN19376-c0-g2-i1, transcribed RNA sequence. HBPM01014491

MSLCTRHCSWKLSDRLRSGWRKTLGEAVEGTVVAMAVCLLLFVDLICTFI
DEIIQNTDLLNPKYEDQGEGVAKWCEHISLVVLVLFMLELSLGVVAFGKR
FFSHIWYLLDFGVVLMSLICEIVSRFYDTDGAQLLAGILILLRAWKFFAF
GFDILMLRHKVHEFEEEHNVNGNEPTPAPALAELDQENRT-

Karenia mikimotoi GISR01074873
Breviolum minutum GICE01010199
Pelagodinium beii HBNF01018002
Symbiodinium sp. HBTG01074859
Durusdinium trenchii ICPJ01008996
Lingulodinium polyedrum JO744110
Selenidium pygospionis GHVN01105692
Gonyaulax spinifera HBNG01058504
Prorocentrum donghaiense GHMW01205561
Dinophysis ovum GKBT01018307

Sar – Stramenopiles
Eukaryota; Sar; Stramenopiles; Ochrophyta; Bacillariophyta; Mediophyceae; Biddulphiophycidae; Eupodiscales; Odontellaceae; Odontella.

Odontella aurita strain CCMP816 TRINITY_DN47737_c0_g1_i1, transcribed RNA sequence. GHBW01044937

MSTTPMKQKSTLKRLQEEEEEAGITTTIRCFVPEKEEVWFVDHNTKEGNW
RRKLIRIVHSTNAQLILGFLLLLDVVFLFTQVFLDAHFPPCYAIIGKAEC
SQGELICNPKPSGIIIVDQILYIGSVSILCVFLLELGLLFVCLQIQFFRN
CGYWLDFVIVLTSLILELVLHGSSDASAASVVIIARIWRLIRIGHGIFNS
KRRQIIARMLADEEDLYIHDHEPYEDSSDEEDYPDKEEEYGSDKVIRRLS
HRIVKRRSYLAKQEDKENGPHL>

Odontella aurita strain CCMP816 TRINITY_DN117390_c0_g1_i6, transcribed RNA sequence. GHBW01194335

MAPQEQGDHFVDESETPSSHSKVGRPPVHIPTDEEVETHVQELHGHDDWR
SKTLHVLHAKPITFTLLGLLVLDVFILFAELYLGAEYPSCTIIERDAISC
CPAEEGAGGDHGGGTHRWLQEMQEIVSDKGFYRDAADKWTSIGSSGKDWS
RALAEETHDDHADGSHAEEAVERLFHDEEDGHGDDHGDEHGDDHGDDHGD
GHSVASASGHGAHGDGHHAHSYCTNGLTDMDPAKYPAACDPHKYEALHHA
HHVLFWLTIAILGTFFLELSIMIVCLGCTFFTKPFYVLDLFVVTTSLLLE
LTFHFVDQESLASLAGLLILARLWRFVRIGHGLVESTNKWQAQKHERLLK
YIDQLEGLCKDNGVELPDRQSIRNLKLLNDEESSTE-

Odontella aurita strain CCMP816 TRINITY_DN105452_c0_g1_i4, transcribed RNA sequence. GHBW01145983

MSPHDSRDSATDVPARPTMQLAGTTSDRKADAKVTALSRSFSVSFNEDPS
EDSNGCFGRKTRRFKPAIPTSEEELDKVEERCGQNSWQMKAVKFINSGPL
QKLLVSLLLCDVIILFIELYLDASYPSCHTVSRDAISCCPAGCSEEKGYV
DKYDDHKDEYDDGHRFLGGSGYGYCEYPLEDTHHPAACDDHKYEGVHKAH
TILFAMTITILSFFMIEILVLIVCLGPMLFFGRFLYVLDFVVIGSSLSLE
IVFATMDDAAAADVAGLLVVFRLWRFVRIGHGLVASTHEMATHKMNKLKK
YTRALEEEVIRCGGVLPESKWSLGLLEETASNHLSSGGDSTSVKKISVEL
GGRVVNTEHLVSKRHTTAGSAEDTERETSSSDVDTKDAFVDNALAA-

Odontella aurita strain CCMP816 TRINITY_DN85551_c0_g1_i1, transcribed RNA sequence. GHBW01102851

<GGASAGHGYCEYPLEDTHYPASCDDHKYEGVHKAHTVLWGITIAILSFF
LLEILTLMVCLGPRTFFCRFLYVLDLFVITSSLALEITFATLDDRKAADI
AGLIVVFRVWRFVRIGHGLVASTHEMAAHKMKKLKKYTRSLEQEVIHCGG
NIPESKWSSQRTLQQLSDNSTTVRDTMPSEDSGREA-

Phaeomonas parva HBGJ01043600 HBGJ01042220
Aplanochytrium stocchinoi HBIN01016972
Thraustochytrium sp. HBSU01005162
Nitzschia palea GJPG01004537
Navicula sp. HBQT01027484
Grammatophora oceanica HBGK01048152
Heterosigma akashiwo ICRV01078048
Chattonella subsalsa HBNW01021031
Thalassionema frauenfeldii HBLL01017232
Licmophora paradoxa HBMT01029076

Sar - Rhizaria

Eukaryota; Sar; Rhizaria; Cercozoa; Chlorarachniophyceae; Amorphochlora.

Amorphochlora amoebiformis, MMETSP0042-doi:10.5281-zenodo.249982-Transcript-23202, transcribed RNA sequence. HBEM01014674

MLGGPRTTNLQHASSDITAYGHQGGMKPEDEENTKVSRAHTQPMVGRERQ
YRRPRRRAPFQSPDGKWRGEIRNDFALGHPILKHFIRPPGRLEGHHGEDW
HHKLNRFLHHHRTHLVLNILLIVDVVLIIIAIELEFAFKNSEIQDLEHAC
EEVEAMHAGASCPSHPGDKSLEDGVRGVEYASVGILCIFAIDNLLLLLAN
GKEFFRNPLYLLDAVVVYLAIIFETVLSGDGGLAGGIIIIVRAWRFVRIG
HGIYETTHDSPQKQDEKSRDITLKRASIGSAPDQKTSPDVENAVKT-

Amorphochlora amoebiformis, MMETSP0042-doi:10.5281-zenodo.249982-Transcript-22745, transcribed RNA sequence. HBEM01014165

<AGSPGRMAVDPFLQATTPLVRPDAKKNVSICSCCMTYATSYHTKADHMG
DCWQRSLLRFLHNAAVQDFLTFLLLLDIICVVSEILIEHYSQEGIEVPED
LELGLKYTSLSILITFCVEIFLYIVAKGLDFFTEPLEVFDMFIVAGSLYQ
DVVYEEATGGLLMLLRVWRFGRIFHGVWATEHERCATRIRQLESKVRYLR
KKNKQLESQKVNLLHHEMYSDDGGSSKAGTMERL-

Bigelowiella longifila HBMK01033472
Lotharella globosa HBIV01019936
Chlorarachnion reptans HBKK01013759
Lotharella oceanica HBHP01002709
Bigelowiella natans HBQC01075387

Haptista – Haptophytes
Eukaryota; Haptista; Haptophyta; Prymnesiophyceae; Isochrysidales; Noelaerhabdaceae; Emiliania.

Emiliania huxleyi, TRINITY-DN7767-c11-g4-i1, transcribed RNA sequence. HBNU01018021

MAEIQTLQPPPTSRLEGGRVKEVHSPEKLERKLKANPRENTLRAKRQAVY
AAMDALEAAGASEVTSPKTRYGARAFGKPLKAQLLSARAEVEKAHAEHGA
DSWQRRCLHLLHSHRVQLFFILLLVLDMLIVITEICLDLEYPSCRLAKRD
TVSCCAAGEEGEHHTLRYLAEAEHGGHHSLCGKGTVEGPHGVGCDEHAHP
AVHTAHAVLTWASVAILSLFEIELLTLLAASGLRDFFSNVYYVLDIVIVS
ASLVLECVFYNTAGLSDLIGLVMFLRLWRLLRIGHAMFASTERASSTDNL
KEVVRELRAELDLLSEWAEEEERASARAPPDDPGVDDIG-

Emiliania huxleyi strain CCMP3266 Ehux3266_tr10784, transcribed RNA sequence. GIZZ01010784

MQSQQRRGAKWVSADSEARALSSRRGSYAWQTKLLAFLHSPRLQALLTLL
LVCDVIAVFGELFIDAEFPSCMYVLRDAIPCCDSGCIGAGDYSAAAAADA
IQTILSLGKSVQGDLHLEHDETRAEICAGQGHSHLDSTGRIGCDSHKHDL
AHKFHKFLFRVSLTVLVVFELELLGLIASLDRAFFRNPLYVLDLIVITVS
LGLETVFRVFSMPEQDLAMALIIVRLWRFVRIGHGIFASTHSVAKEKSDK
LHAEIRALEEEYTRALNQAASLATTTRPTRLASAPSSTSGSPSATRPPPF
RGRSASEASAATHDLQGAGGEKLADGVAAALSQRSNTPTSPVGAPAMRRP
PAAAPLGAAHRALQLGPSSSAARTGMPGVGTRMGAGEAAAAERQAPPAAE
PEPAEGAAAAESVELELDALDMST-

Calcidiscus leptoporus HBER01001684
Gephyrocapsa muellerae HBRT01056448 HBRT01109117
Coccolithus braarudii HBEY01039257
Scyphosphaera apsteinii HBMI01021482
Gephyrocapsa oceanica HBTM01082732 HBON01067641
Prymnesium parvum GBYI01026351
Phaeocystis sp. HBQW01022812
Phaeocystis globosa HBRY01039040

Haptista - Centrohelida
Eukaryota; Haptista; Centroplasthelida; Pterocystida; Raphidista; Choanocystis.

Choanocystis sp. FB-2015 c53027_g1_i1 transcribed RNA sequence. GDKX01062209

MEHNPQRDSLRQPISEKYAAKRASHGSLEARLVDTFKMLDRNANGLVNVN
ELRVLVNTYLETPLETEEIVDTLQQLLPDKGLAPDPHKSIKLNRDDFLKA
MIHFREQNSFPTPLMTRLHSDPITFNGRSALSTSKPCLSERQEHHVVNIS
ASNPNLEEHRQMTRKSTRQRLSRALLNLPDDLYVSGETTLPSKSQSGRSS
HSASLAELVEEPENPKQEAKIENRDRSARVALAQHLNSTAMQVLVVFLVL
VDAFVVIAELMVLSMPCHVPEPCGLSNFNATETLDGFGQCLSKYEHTCES
FHSLMSVLRWSSKGILMFFLAQIICLALCVGWRFFKQPFFVMDLLVVSSA
MALEFSVSLRGGRMIVFVLLWRFVRIAHALATTIDIHISNMERRVMRQTG
ADVRAASTLQREIRSHQQTNRAVMERIQANSNRIANEEEFIESLKMEELR
DMILRERNHTSEVKQRVKELQEILTVVQGHMEAKRSQFKNRHIKHQARQH
PGLAVQYSTSSSRSVSTAGTIHHRDPVV-

Choanocystis sp. FB-2015 c20243_g1_i1 transcribed RNA sequence. GDKX01034169

MWVEGKGVESLEGRLKETFKMLDKDSDGHVDVHDIKVLLELRDQQPRTDA
EVIAIINRILPGKTHLAALTRKGKVTVSEEEFVLAMLHFLSNEATERAID
HDDLHNAFIDHLLDLPESDQSVTIDVDKSSKASRKSFAAARKSFAHAGQA
VKQARKSFANMALATRDAELDSDRAKAAGLAGFLNARVVVASRLNSTPMQ
ALIVLLVILDAFAVAMEIVLLGVQCKTPSECYAPSAEVHHKAGGGVVTDG
SKYDATTCLHKFDHVCHAVHQIEYILKWISKGILLFFIAQISILAVSIGI
RSFLRQPFFVLDFVVVSVALGLEFGLKNAGGALLGFVMLWRLIRIVHAFA
TTLDVHKGTVERHILRQEKAEAKEALQIQRAIQEHRKLGQPLVNAIRHKT
IRRIEKRSALGKSPRLSAMTNESGLAATPVSHNMSVSSATTLPDISITLV
ETEPEVRDEEYWMGRVATLEAELKEDKVHVRELESKFVDIQNTLTTVYAH
LEGKKQRFLEKVLKQNQALNIPARGSLLEFTKEMQQIQPK-

Amoebozoa - Discosea

Eukaryota; Amoebozoa; Discosea; Flabellinia; Dactylopodida; Paramoebidae; Paramoeba.

Paramoeba aestuarina, MMETSP0161-doi:10.5281-zenodo.249982-Transcript-29362, transcribed RNA sequence. HBKR01021517

MGVIKGGLYSTEGMWKHNSTKRDKVLHVIHHKYTHFFITALLVIDLCVVI
TSISLEIEYLTSEVTDLEECVHECFFGDEHDEEHRSLTQQSFRQSNHETQ
EKNTNEALKRREERERERSEEYRESRQNDDSDHDEEKSEKFLECEATAMFE
DDDDNWGNGNLKEAEEALAYVSIAILCVFIIEHMILFGAMRMDYLRSPIL
IFDFFVIAVSLALEIIFQGQPEAGLLIVARAWRFIRILHGFHESTSDEVV
KETVHALNSKREDILNIYAALDDRLLVGASKKEAMENVAEQYPETIFEIL
QILGHHFQKDKQEHLHIKDTIEHRMHPTPSRTHL-

Balamuthia mandrillaris strain CDC-V039 BamaA010555t1, transcribed RNA sequence. GISS01003879

MAEHGDATPLLPSHGREAIGNGNHSRWRWLLDYPHQRKVKRETAAGPPSG
LRALRLSMARFLEGRFMQYFLLALLVLDVLVLVVELGIMEASCESGKEKS
DHTFHIIEQVLRYVTLSILSIFAFELLLLLLALGLDFLKHPLYIVEVAII
ATAFVLEIGLRHLQAISGLLVIFRLWRLLRLVHGVITAQQDLHRNTKQNL
EESRARVHELEQEVERLRRRGSLRD-

Balamuthia mandrillaris strain CDC-V039 BamaA008510t1, transcribed RNA sequence. GISS01013796

MTTRRTSSGRREKDEGAHELERIDHHHQEEEEKGESSEGKDEEAVNPLGK
TKQKHLREQHHKYQHCKHPHHHHLLETTRRGLVGKFGWLLGTHKYPHQKK
RERLEKAEHFPKIHRIRVKIAQFLEDPRFQYFLVALLLVEVVILVVELLL
LERTCEGEEENEEEDHAVHVVEEVLFWFTVTILSIFALELLTLFFALGLD
FIRHPLYVVDALIVAAAFVIEVVLRDTLAGSLLLMLRLWRIIRIAHGIIT
AHQELHHQTKKDLHVALHRINELEKLLAEA>

Paramoeba pemaquidensis GEWA01008129
Balamuthia mandrillaris GISS01013796
Tubulinea HBYL01016471
Acanthamoeba sp. IACY01017688
Acanthamoeba castellanii GJZG01027302
Vannella HBXS01019840

Amoebozoa - Mycetozoa
Eukaryota; Amoebozoa; Evosea; Eumycetozoa; Dictyostelia; Dictyosteliales; Raperosteliaceae; Raperostelium.

Raperostelium potamoides strain FP1A RPO_TRIN_CL14637, transcribed RNA sequence. GIOX01005152

MALVKGRYKFPFHFAKNSPRHKARRLWRRKLGSFLESNRVQIAIVALIFL
DLIIVIIELFLEEHYKSQCAEEHEIPHVIHRLENALGIITLVLLGIFEFE
ILLLLFAFGRDFFKHPLYVFDAIVITVSIIVEVVFRDTAGALLVVFRLWR
VVKIGHGIAISVETHDKKKYKELKSRYRKKVEDNKVYLNQINVLRNRMGE
IAISPPNLSEVSANTSSIFSPSRSNEIQSPNSTTPINEIKDDISDLSADS
DEDNFNEKLKINSNNNNP-

Hagiwaraea rhizopodium GIOY01027566
Raperostelium gracile GIOO01028008
Physarum polycephalum GDRG01015357
Heterostelium gloeosporum GIPA01000642
Cavenderia multistipes GIOI01021321
Tieghemostelium menorah GIPF01006404
Cavenderia bifurcata GIOH01013395

Cryptista
Eukaryota; Cryptophyceae; Pyrenomonadales; Chroomonadaceae; Chroomonas.

Chroomonas sp. Dc01 RNA, 13hcomp50078_c1_seq1.p1, mRNA sequence. ICPR01036203

MMNAPYFPQVGMGFGQHPPPFGLSVDGGEKHEHHAEAQAAHDAAESLVHK
LRTHGERLLKSGARRFRPIRSYWLTADQERDRVIEKHGEDSWQAKVVDFI
QSRSVQALLISLLILDVIIVAIELFLEAEYPPCVIVKRDAVSCFNATALV
GMSAAALESSTHARAKILSPRGEGTLKHAAAATRTEALHTHTPENGTVHE
GVVEGGEHHDVCEEAGLISTGFEASCDKHKWSRVHDTHTTFLWISVAILV
AFLTELLALLACLAFDFVRNPLYLFDLVIVLVSLVLEIVLEHVGEVQLSA
LSGLLVFARIWRFVRIAHGLATSVHESEAASHEEITKQAEELHKQVVELR
AKLAGSARRGLMAR-

Geminigera cryophila HBHS01020640
Teleaulax amphioxeia GKBS01005298

Discoba – Euglenozoa
Eukaryota; Discoba; Euglenozoa; Euglenida; Spirocuta; Euglenophyceae; Euglenales; Euglenaceae; Euglena.

Euglena gracilis comp31701_c0_seq1 transcribed RNA sequence. GDJR01054876

```
MSYFDDTFVGAPAPSVEATKVEDGCPIDCHKLASPSKAPTLRQKVHHFLS
CRPFKIFMMALLLLDLILVMASVLLETGSLQVSLDECERSIHDCEHICSN
STDFTATATNYCTNTHGQTCHYNHPSLATALHLAEKVLAYISIGLLSFFL
LERLVGIICEGLKFFSCAFKVFDFVVIIVSLVLEILFLGQPGVGLIAIGR
FWRFVRIGHGFHEAEELTHPVDEFVKEHKAALFALHRRLLGVPGLYGTEG
AVLAENAAAVREALALLEQDGFGGLLLGIADAAVAGHRQHQEKEQLHYWK
LQ-
```

Euglena longa GGOE01032297
Neobodo designis HBGF01052964

Figure S2. Alignment of H_V1 and H_V2 from Xenopus.

```
Query  1    MAGCLRHFTSVGDDTKKREWKEEDVEVAHEEEKKNTPHPFIASYSLRGALKWLFSSHKFQ  60
            MAGCLRHFTSVGDDTKKREWK+EDVEVA+EE  KNTPHPFIASYS RGALKWL SSHKFQ
Sbjct  1    MAGCLRHFTSVGDDTKKREWKQEDVEVAYEEPLKNTPHPFIASYSFRGALKWLLSSHKFQ  60

Query  61   IVIISLVILDALFVLVEVLLDLELLAEKVDHIIPEIFHYLSVSVLSFFILEIAGKLYAFR  120
            IVII LVILDALFVLVEVLLDLELLAEKVDHIIPEIFHYLS+SVL+FFILEIAGKLYAFR
Sbjct  61   IVIICLVILDALFVLVEVLLDLELLAEKVDHIIPEIFHYLSISVLTFFILEIAGKLYAFR  120

Query  121  LEFFHHKFEVFDAAIVVISFIIDIVYISREDIFNAVGLLILLRLWRVARIVNGVIVSVKS  180
            LEFFHHKFEVFDAAIVVISFIIDIVYISREDIFNAVGLLILLRLWRVARIVNGVIVSVK+
Sbjct  121  LEFFHHKFEVFDAAIVVISFIIDIVYISREDIFNAVGLLILLRLWRVARIVNGVIVSVKT  180

Query  181  RAEEKIHKLEENQRSLLEKVTQLEQQSAQQEQEIARLQKLLKQHNVFPDS  230
            RAEEK+HKL+E + SLLEKV QLEQQ AQQEQEI RL KLL++HNVFP S
Sbjct  181  RAEEKMHKLKEQKGSLLEKVAQLEQQCAQQEQEIGRLHKLLQEHNVFPAS  230
```

Figure S3. Cephalochordata sequences

Opistthokonta – Animalia – Cephalochordata
Eukaryota; Metazoa; Chordata; Cephalochordata; Leptocardii; Amphioxiformes; Branchiostomidae; Branchiostoma.

Branchiostoma belcheri voltage-gated hydrogen channel 1-like (LOC109464640), transcript variant X1, misc_RNA. XR_002139895

```
MDKLKNAFKFELFQNDDSSSVITSSDATSSSGNAEPKTFREKLLHVLHST
EFQVAVVILVIVDCILVVFELLIDLGGIKLCEEAVRAECESAGTTATMTP
```

AEEAEKEAECDHPAPEILHYMSIAILTIFLIEIMFKVYAYQKDYLKHKME
LFDAVVVIISFCFDVAYANHEDAFDGIGLLVVLRLWRVTRIINGILMSVQ
HTAEKKINAHKQARQEVQEELNKMIAHAHDLEKEIDLLRKTLRENGISVE
SIPRTPEVSSSQVKVEAEITPTTEYATPAHFSAFSGQDDTQA-

Gene:
Branchiostoma belcheri isolate BF01 breed outbred unplaced genomic scaffold, Haploidv18h27 scaffold1, whole genome shotgun sequence.
NW_017802379

Branchiostoma belcheri uncharacterized LOC109463761 (LOC109463761), mRNA. XM_019760615

MTPCDGEKKPPPSGEATVGGIFPDEIAVIKKQAQNDKRRPLKIVIIAGVA
VMAVLAIGVLLLITRPSRHGVVTCSLNFWTGKQLLHETVETDKDAETDAF
YTEGSRGEAAVMYDHSSMMKAFKLSRSNKTCFIFEETQGEKNAVKKTAEE
LEEKQDGSLQFAEYGGAMLMTVDTERPARPVLSQKLQNFCGQLEPRWAKL
TPATEEEQQGDRVEIIMPAEARDMGELAEMPVGPGNEKETPPTPSSTHPL
TESRDHHHDNQDCRHKLKHMLERQSVHIAIVVLIVLDTLIVIMELLIDVR
VIKLCPDPPDVCVPKAGHNGTTGLVTTGAPGHHVIDAGDHGTGGHEECHH
VLIEVLHVVSILILCIFVVEIALKIYVDRLEFFKNGFHVLDAVVVLVSLG
LDIASLVRPSAFTDAGGLLILLRLWRITRIVNGIIISVEEEWEHKVNHLK
HEHQLVERERDRLLKENALLQKTLTNHGIDIPKLPPDSGDESTCEFEFE-

Gene:
Branchiostoma belcheri isolate BF01 breed outbred unplaced genomic scaffold, Haploidv18h27 scaffold1, whole genome shotgun sequence.
NW_017802379

Branchiostoma belcheri uncharacterized LOC109467017 (LOC109467017), transcript variant X1, mRNA. XM_019764911

MPRFKGQQQHHETCRHLAVEDVMVADELGLGMSSADLYNRNVEGDFLDLE
AAIFCDIEEEKTCKQRVQQLLDGPATQITIVLTSWLLSHVLLLELLVDLS
AIHFHHDKMVARIIHWVGLGVLSVFTVEVLTRLVCHQMQFFDKKIEVLDL
AVVIIACVPMIVVSVELAPSTAWDGFSLVIILRIWRCYRVVQGCVSPVRE
EASRKVHVLLQAQRRAHQELQTLYLMHDENQEEIHRLRLLLGRREAEEDN
VSQQLQVALERKDSQYVAQLIHTIEQRQGRNKGRQRDGGRDSPYSTIVVH
AHQLSDSNDVPSGTIASRLSESTTGDSGICEETRPQQQTNDESRNHANSS
GVTQLPKCGQQALTVVEPRGSHQQHSNRSPCSNSKRNPAVSSCDMDRIDV
IGSEIILQHRNDSTGKKFLDGKLPSNKDSRLLFRDNKSMPSNGKVSHSKN
KVSEIKVNKSLDDDHKKAVSMDNKTVSKNNTATAQTNGKVRKGKKKAGSG
DSYVNGALLLEMTELQQKGDTGYCNEIVIEQFYQNGNAPATAL-

Gene:
Branchiostoma belcheri isolate BF01 breed outbred unplaced genomic scaffold, Haploidv18h27 scaffold173, whole genome shotgun sequence.
ACCESSION NW_017803191

Figure S4. Chelicerata sequences

Eukaryota; Metazoa; Ecdysozoa; Arthropoda; **Chelicerata**; Merostomata; Xiphosura; Limulidae; Limulus.

Atlantic horseshoe crab

Hv1:

Limulus polyphemus voltage-gated hydrogen channel 1-like (LOC106464594), transcript variant X1, mRNA. XM_013924749

MEDTTEKTGDSTSIATNLESDPDIQPLITFRERLTKLLHSYKFQVGVITL
VIVDCLLVISELLVDLNILSVNSHSSAPHVLHYLSISILSLFIIEIGAKL
YAFRLEFFHHKLELFDAVIVLVSFALDITFRDKESAVSGVGLLIILRLWR
VARVLNGIVLSVKTQADHKLAKEQKKRENLEQELARSRDYIAALEEEVET
LRRILKDNNIKELPPTVIDNGAFKCTTLNVVAEVNHMIT-

Gene:
Limulus polyphemus unplaced genomic scaffold, Limulus_polyphemus-2.1.2 Scaffold1698 Sequence ID: NW_013667308.1

Hv2:

Limulus polyphemus transmembrane protein 266-like (LOC106463214), mRNA. XM_022390662

MRNSDPLVWGLTGDTSLDDDGPTRLCEKIWATVNGKVFSAVIVTLVFVEG
LVIFSELLIDFEVVQDPQWKTSLNCSANVSGLVFSSAGANSPKLRMAKDI
LSYISIVILIIFVFEVGFRLVSGRARYLIQGMEICDAIVVLVAFGLDIAF
LTTPSKKGAGKEAAVLIILLRLWRIKRILQSVIDNTRLEMGHFLSICERE
KIQAEHKVDILILKVEDLEHEVAYLKEKLKKTEKESLYAKRQRKKDGYSS
TQHKHPTITIGVETSPARHPCTGTQTAIVICEQHIPEEKVTKEQVMDMRT
FADVTSTRIIADALCMVTGNPNQFLRSPTTGGSTVKDGTTSHCRIAGDFE
SGYISNVSGITWDKAASRIPTLGTRTTLKCPESSPESGYGSSSSARNPAA
SSVSPLDTGTETASTSTGSSKHTDTVFLFPDPTGQRREVELGVEMDILEE
ISEIERVKHIEFDPNKQDQDIPMTSL-

Gene:
Limulus polyphemus unplaced genomic scaffold, Limulus_polyphemus-2.1.2 Scaffold1350 Sequence ID: NW_013666960.1

Hv3:

Limulus polyphemus uncharacterized LOC111087200 (LOC111087200), transcript variant X1, mRNA. XM_022393049

MRNFSMFFNSKEELMRNSEPLVWSLTGDESLDDDDSTRLCERIWVSVNGK
VFNAVIVTMVFVEGLVVFSELLIDFEIVQDPHWKTIRNCSANVPALIFPS
SEAVSPNLRNTKDVLSYISIVILIIFVFEIGCRLAVGRAKYLIQGIEICD
AAVVLVAFGLDIAFLTTPSKKGAGKEAAVLIILLRLWRIKRVLQSVIDKT
RVEMGHFLSICEREKAQAEQKVDILILKVEDLEHEVAYLKEKLKKSEKES
LCAKRQRKKEGYSGIQQKHPKITVGVETSPARHPCTGTQTARAICEGFIL
DEKATKRKVMDITTFAEITATRIIAKALCVASPDSGQYVRFLATKDAVGI
SGTTLCRRIDGSFESGYGSNTSGMTLDKTTNCIPSVGIRRTVKCPESSPE
SGYGSSSSARNTVATSVSPLDIEMETTSVSTGSTKQTDTVFLFPDSANQC
RKAERGVEMDILEELSEIERIKQVEFDPNKQDEDIPMTSL-

Gene:
Limulus polyphemus unplaced genomic scaffold, Limulus_polyphemus-2.1.2 Scaffold1865 Sequence ID: NW_013667475.1

Figure S5. Sequence alignment of two putative *Oikopleura dioica* H_V channels with *Ciona intestinalis* H_V1

```
                ....|....| ....|....| ....|....| ....|....| ....|....|
                        10         20         30         40         50
CiintHv1        MEGDNCNKSR HKSHNMINPN YASVRCTQPL PSVIQLRSRN KMIGITEDPS
OidioHvL1       ---------- ---------- ---------- ---------- ----------
OidioHvL2       ---------- ---------- ---------- ---------- ----------

                ....|....| ....|....| ....|....| ....|....| ....|....|
                        60         70         80         90        100
CiintHv1        SDSEPVSSNQ PLLLTNLSYE VHTFNDNNNH ERPAPQEQST QNTMISMQSE
OidioHvL1       ---------- ---------- ---------- ---------- --------MS
OidioHvL2       ---------- ---------- ---------- ---------- --------MD

                ....|....| ....|....| ....|....| ....|....| ....|....|
                       110        120        130        140        150
CiintHv1        QKSDRFTASN LGMFQYMKFE IGEDGDDHEE EAILTNREKL RHILHSKPIH
OidioHvL1       ENSSVVESTP ---------- -FLEILKSDM TCWEKTSRVV HKLLHSHTTQ
OidioHvL2       NRISKFFSS- ---------- ---DTEAYLE RTPVTISEKI AHFLHSSTCH

                ....|....| ....|....| ....|....| ....|....| ....|....|
                       160        170        180        190        200
CiintHv1        VAIIVLVVLD SFLVVGELLI DLKVIIVPHG ---------- ----------
OidioHvL1       AILLFLVLVD CAVIACEIVF DEKVKTHNDY CGANSTLCQE EIDNEQTKKW
OidioHvL2       LIVIVLVLVD CAFVLTELIF DGKLDTYAEY C---DPTPSC SEQSHKLHHW

                ....|....| ....|....| ....|....| ....|....| ....|....|
                       210        220        230        240        250
CiintHv1        NPAPEILHGF SLSILSIFMV EIALKIIADH RHFIHHKVEV LDAVVVVISF
OidioHvL1       KKIYKALYYT SLTLLSVFVV EFILKIVFSA KKFLKSWVHI FDALIVISSW
OidioHvL2       EHVTHVIHIC SIIVLTIFMI EVILKVIYTR EHFFSHKIEM IDGVVVLLSW

                ....|....| ....|....| ....|....| ....|....| ....|....|
                       260        270        280        290        300
CiintHv1        GVDIALIFVGES-EALAAIG LLV---ILRLWRVFRIINGIIVTVKTKADDRVH
OidioHvL1       ILMLIMLNKDVKNGMIAEFL IAF--------RVIRLIHGMKEALEEANELLHE
OidioHvL2       ILDLIMLNPNLV-GKWAEFI IIF--------RFLRLINGMILSAKQAADKRVH
CiintVSP        VVTIFYTVLDEYVQETGADG LGRLVVLARLLRVVRLARIFYSHQQMKASSRRT

                ....|....| ....|....| ....|....| ....|....| ....|....|
                       260        270        280        290        300
CiintHv1        GVDIALIFV GES-EALAAI GLLVILRLWR VFRIIN--GI IVTVKTKADD
CiintVOSP       VVTIFYTVL DEYVQETGAD GLGRLVVLAR LLRVVRLARI FYSHQQMKAS
OidioHvL1       ILMLIMLNK DVKNGMIAEF -----LIAFR VIRLIH--GM KEALEEANEL
OidioHvL2       ILDLIMLNP NLV-GKWAEF -----IIIFR FLRLIN--GM ILSAKQAADK

                ....|....| ....|....| ....|....| ....|....| ....|....|
                       310        320        330        340        350
CiintHv1        EIKKKNSELE LQIHNLEEKL SQKEQDMSRL HEILRCNNID IPPTVPLTTS
OidioHvL1       NMEEEIHALK AENNKLKEEL LAVKDR---- ---------- ----------
OidioHvL2       EQKRKVQELE KELVDLKKQY ETQNNQ---- ---------- ----------
```

```
              ....|....| ....
                      360
CiintHv1      VQIHSTTTAS ADV-
OidioHvL1     ---------- ----
OidioHvL2     ---------- ----
```

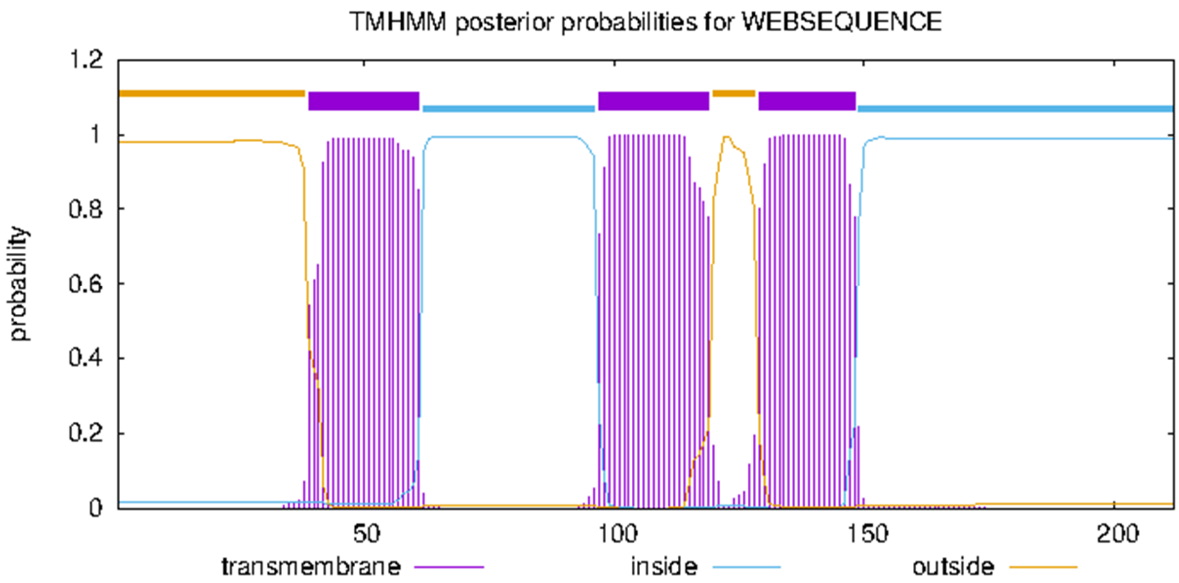

Transmembrane prediction using TMHMM of *Oikopleura dioica* sequences identified only three transmebrane spanning segments.

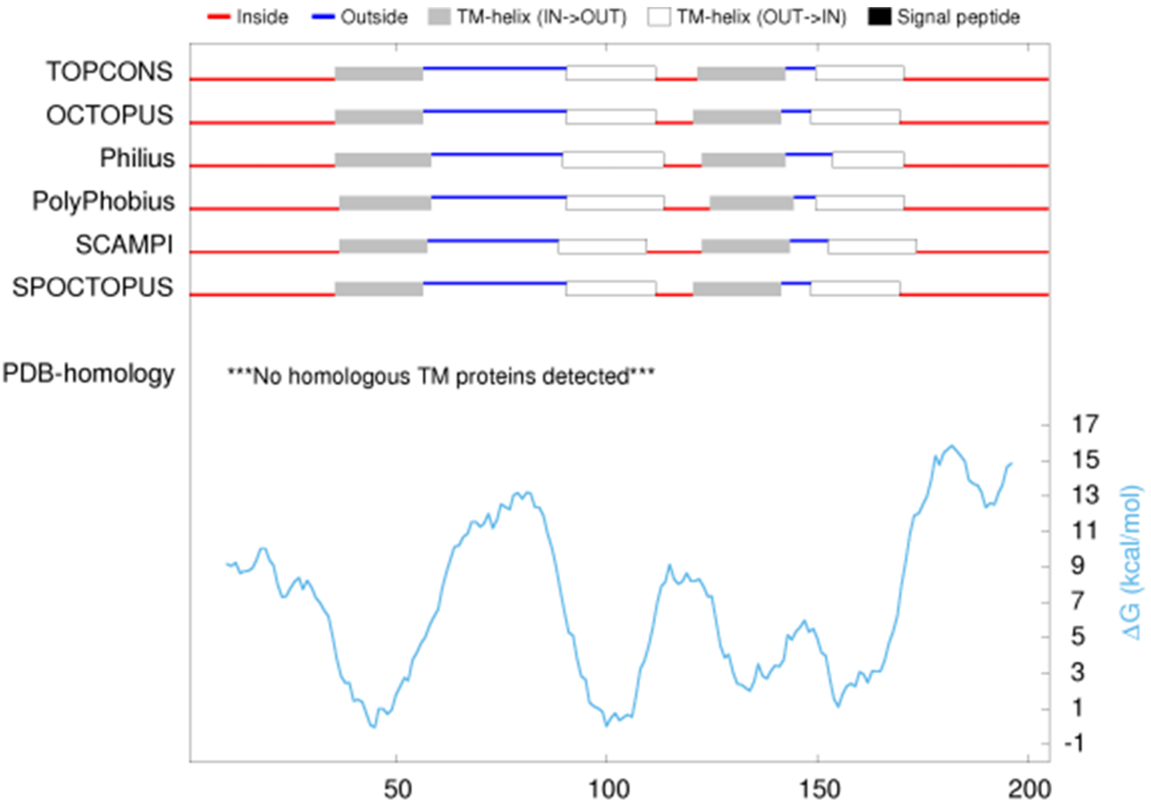

High-resolution image

Consensus prediction (TOPCONS):

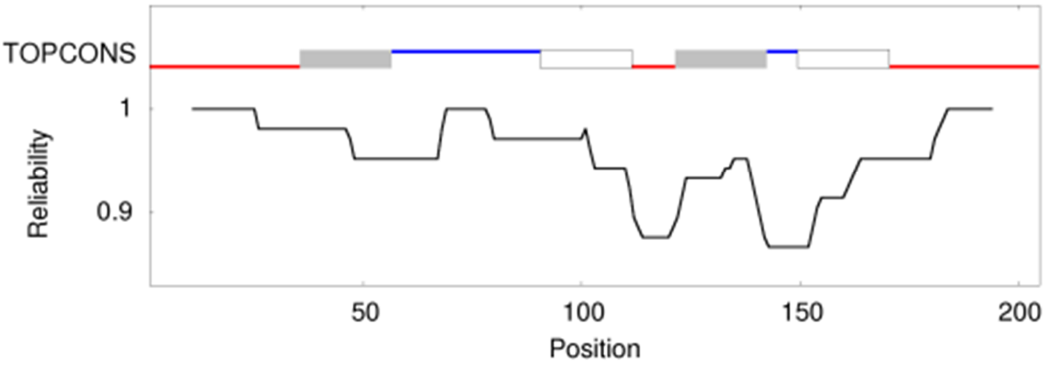

High-resolution image

Predicted signal peptide and TM-helix positions (position starting from 1):

```
TOPCONS       TM1: 36-56, TM2: 91-111, TM3: 122-142, TM4: 150-170
OCTOPUS       TM1: 36-56, TM2: 91-111, TM3: 121-141, TM4: 149-169
Philius       TM1: 36-58, TM2: 90-113, TM3: 123-142, TM4: 154-170
PolyPhobius   TM1: 37-58, TM2: 91-113, TM3: 125-144, TM4: 150-170
SCAMPI        TM1: 37-57, TM2: 89-109, TM3: 123-143, TM4: 153-173
SPOCTOPUS     TM1: 36-56, TM2: 91-111, TM3: 121-141, TM4: 149-169
PDB-homology  ***No homologous TM proteins detected***
```

Sequence and predicted topologies: (i: inside the membrane, o: outside of the membrane, M: membrane region, u: non-membrane region but location unknown)

```
               1                               41
Seq.           MDNRISKFFS SDTEAYLERT PVTISEKIAH FLHSSTCHLI VIVLVLVDCA
TOPCONS        iiiiiiiiii iiiiiiiiii iiiiiiiiii iiiiiMMMMM MMMMMMMMMM
OCTOPUS        iiiiiiiiii iiiiiiiiii iiiiiiiiii iiiiiMMMMM MMMMMMMMMM
Philius        iiiiiiiiii iiiiiiiiii iiiiiiiiii iiiiiMMMMM MMMMMMMMMM
PolyPhobius    iiiiiiiiii iiiiiiiiii iiiiiiiiii iiiiiiMMMM MMMMMMMMMM
SCAMPI         iiiiiiiiii iiiiiiiiii iiiiiiiiii iiiiiiMMMM MMMMMMMMMM
SPOCTOPUS      iiiiiiiiii iiiiiiiiii iiiiiiiiii iiiiiMMMMM MMMMMMMMMM
PDB-homology

               51                                          91
Seq.           FVLTELIFDG KLDTYAEYCD PTPSCSEQSH KLHHWEHVTH VIHICSIIVL
TOPCONS        MMMMMMoooo oooooooooo oooooooooo oooooooooo MMMMMMMMMM
OCTOPUS        MMMMMMoooo oooooooooo oooooooooo oooooooooo MMMMMMMMMM
Philius        MMMMMMMMoo oooooooooo oooooooooo oooooooooM MMMMMMMMMM
PolyPhobius    MMMMMMMMoo oooooooooo oooooooooo oooooooooo MMMMMMMMMM
SCAMPI         MMMMMMMooo oooooooooo oooooooooo ooooooooMM MMMMMMMMMM
SPOCTOPUS      MMMMMMoooo oooooooooo oooooooooo oooooooooo MMMMMMMMMM
PDB-homology

               101                                         141
Seq.           TIFMIEVILK VIYTREHFFS HKIEMIDGVV VLLSWILDLI MLNPNLVGKW
TOPCONS        MMMMMMMMMM Miiiiiiiii iMMMMMMMMM MMMMMMMMMM MMoooooooM
OCTOPUS        MMMMMMMMMM Miiiiiiiii MMMMMMMMMM MMMMMMMMMM MoooooooMM
Philius        MMMMMMMMMM MMMiiiiiii iiMMMMMMMM MMMMMMMMMM MMoooooooo
PolyPhobius    MMMMMMMMMM MMMiiiiiii iiiiMMMMMM MMMMMMMMMM MMMMoooooM
SCAMPI         MMMMMMMMMi iiiiiiiiii iiMMMMMMMM MMMMMMMMMM MMMooooooo
```

```
SPOCTOPUS       MMMMMMMMMM Miiiiiiii MMMMMMMMMM MMMMMMMMMM MoooooooMM
PDB-homology

                151                                            191
Seq.            AEFIIIFRFL RLINGMILSA KQAADKRVHE QKRKVQELEK ELVDLKKQYE
TOPCONS         MMMMMMMMMM MMMMMMMMMM iiiiiiiiii iiiiiiiiii iiiiiiiiii
OCTOPUS         MMMMMMMMMM MMMMMMMMMi iiiiiiiiii iiiiiiiiii iiiiiiiiii
Philius         oooMMMMMMM MMMMMMMMMM iiiiiiiiii iiiiiiiiii iiiiiiiiii
PolyPhobius     MMMMMMMMMM MMMMMMMMMM iiiiiiiiii iiiiiiiiii iiiiiiiiii
SCAMPI          ooMMMMMMMM MMMMMMMMMM MMMiiiiiii iiiiiiiiii iiiiiiiiii
SPOCTOPUS       MMMMMMMMMM MMMMMMMMMi iiiiiiiiii iiiiiiiiii iiiiiiiiii
PDB-homology

                201
Seq.            TQNNQ
TOPCONS         iiiii
OCTOPUS         iiiii
Philius         iiiii
PolyPhobius     iiiii
SCAMPI          iiiii
SPOCTOPUS       iiiii
PDB-homology
```

Figure S6. Multiple alignment of H_V channels.

```
         ....|....| ....|....| ....|....| ....|....| ....|....|
                 10         20         30         40         50
hsHv1    ---------- ---------- ------MATW DEKAVTRRAK VAPAERMSKF
ciHv1    ---------- -MEGDNCNKS RHKSHNMINP NYASVRCTQP LPSVIQLRSR
spHv1    MAEGEGEGRC AVDSGTMKPD LKQTEEVSQG LLEQGDGGSS SHGNNKVGER
ehHv1    ---------- ---------- ---------- ---------- ----MAEIQT


         ....|....| ....|....| ....|....| ....|....| ....|....|
                 60         70         80         90        100
hsHv1    LRHFTVVGDD YHAWN----- ---------- --INYKKWEN EEEEEEEEQP
ciHv1    NKMIGITEDP SSDSEPVSSN QPLLLTNLSY EVHTFNDNNN HERPAPQEQS
npHv1    ---------- ---------- ---------- ---------- ----MWLKMD
etHv1    ---------- ---------- ---------- ---------- -MGPNEVELG
spHv1    SGDGQFAEQG GVCETSEDKN DGCETHSGAK TDKHKTKLEG ETAPADAEMF
acHv1    ---------- ---------- ---------- ---------- ------MKLD
acHv2    ---------- ---------- ----MRMSRS IEYPSEKNGE PSCIEAEQRS
aoHv1    ---------- ---------- ---------- ---------- ------MASP
amHv1    ---------- ---------- ---------- ---------- -----MIDAR
kvHv1    ---------- ---------- ---------- ---------- ------MDRI
kvHv2    ---------- ---------- ---------- ---------- --------MW
ehHv1    LQPPPTSRLE GGRVKEVHSP EKLERKLKAN PRENTLRAKR QAVYAAMDAL
ehHv2    ---------- ---------- ---------- ---------- ------MQSQ
egHv1    ---------- ---------- ---------- ---------- ------MSYF


         ....|....| ....|....| ....|....| ....|....| ....|....|
                110        120        130        140        150
hsHv1    PPTPVSGEEG RAAAPDVAPA PG-------- --------PA PRAPLDFRGM
ciHv1    TQNTMISMQS EQKSDRFTAS NLGMFQYMKF EIGEDGDDHE EEAILTNREK
npHv1    AHKRLSEDLE KVIMKEDG-- --------NS SIMTEPDHNI QP-SKTVRER
etHv1    TLS--SDASG PLDRHKQ--- ---------- ---SRKTQDG FERIKTFREK
spHv1    GFRRLSDTTK PSEGNDQQRV IVKDDSSDSV VSDSHDGHPA RTEPLSLREK
acHv1    GLRKMQDDLV KVIERDD--- --------TS TVTSDSDETI ARGPKTLRET
acHv2    GETKMLKSEQ SQDEAETSDW SE-------- ---NEDSHSG KLDANSCKGK
cgHv4    ----MENSEE FREGTEN--- ---------- ---------- -----GCKAK
aoHv1    SDPLLHEHTG PRSLRQRP-- ---------- --IYLPEEQG QRIIAQWRRA
```

```
amHv1    TRRSSMDDQL PADELKEMGQ PGTTAAVMAS TVCLTEDDTE CPANKNHREK
kvHv1    LHHAVHTVHT SKSARDAEG- ---------- ---------- ---HGTWQSK
kvHv2    RTASATGTKK SKIRRIVP-- ---------- -------HIL DEISHEATHF
ehHv1    EAAGASEVTS PKTRYGARAF GKPLKAQLLS ARAEVEKAHA EHGADSWQRR
ehHv2    QRRGAKWVSA DSEARALS-- ---------- ---------S RRGSYAWQTK
egHv1    DDTFVGAPAP SVEATKVEDG CP-------- --IDCHKLAS PSKAPTLRQK

         ....|....| ....|....| ....|....| ....|....| ....|....|
                160        170        180        190        200
hsHv1    LRKLFSSHRF QVIIICLVVL DALLVLAELI LD-------- ----------
ciHv1    LRHILHSKPI HVAIIVLVVL DSFLVVGELL ID-------- ----------
npHv1    LRKLLHSHKF QISVITLVII DCLLVITELL ID-------- ----------
etHv1    AKLLLESPKF HIVVLTLVII ELTMVVAELM ID-------- ----------
spHv1    LHEIMETQKF HIAILVLVVI DCILVIVELV IDFEVLSANN HVPEAGCAHP
acHv1    LDDVIHSQKF MVFIIVLVVL DCLMVIAELL FD-------- ----------
acHv2    LAAFLKTNLV QYSIIALVIL DCLIIVMELL IDMNIIVFPE DDPHHPPGEG
cgHv4    MRKVFHNPIT HLIIIAFIVL DIAILLVVLL VDINVIQVRA ETEED-----
aoHv1    ARDFLSSRRG HYLVLLLVSV DVACTFADFL IELHVCELT- ----------
amHv1    LNELLHGDKI QYAIVVLVII DIIIVIAELV LD-------- ----------
kvHv1    LNEALNSSKV HTILNVLLIC DLMTVIIGML LEQYYSDSQV QGLTEAFKDC
kvHv2    RRLLLHSKPV HFTILVLLIA DGLICLTCGV LEAHYLHGKS DDCQNYVNKC
ehHv1    CLHLLHSHRV QLFFILLLVL DMLIVITEIC LDLEYPSCRL AKRDTVSCCA
ehHv2    LLAFLHSPRL QALLTLLLVC DVIAVFGELF IDAEFPSCMY VLRDAIPCCD
egHv1    VHHFLSCRPF KIFMMALLLL DLILVMASVL LETGSLQVSL DECERSIHDC

         ....|....| ....|....| ....|....| ....|....| ....|....|
                210        220        230        240        250
hsHv1    ---------- ---------- ---------- ---------- ----------
ciHv1    ---------- ---------- ---------- ---------- ----------
npHv1    ---------- ---------- ---------- ---------- ----------
etHv1    ---------- ---------- ---------- ---------- ----------
spHv1    QE-------- ---------- ---------- ---------- ----------
acHv1    ---------- ---------- ---------- ---------- ----------
acHv2    SSHHPVAFAS RSSNLTGDNH TVYPAHHIH- ---------- THHDNSSNLT
cgHv4    ---------- ---------- ---------- ---------- ----------
aoHv1    ---------- ---------- ---------- ---------- ----------
amHv1    ---------- ---------- ---------- ---------- ----------
kvHv1    LEKRTFCP-- ---------- ---------- ---------- ----------
kvHv2    --100aa--- ---------- ---------- ---------- ----------
ehHv1    AGEEGEHHTL RYLAEAEHGG HHSLCG---- ---------- --------KG
ehHv2    SGCIGAGDYS AAAAADAIQT ILSLGKSVQG DLHLEHDETR AEICAGQGHS
egHv1    EHICSNSTDF TATATNYCTN THG------- ---------- ----------

         ....|....| ....|....| ....|....| ....|....| ....|....|
                260        270        280        290        300
hsHv1    ---------L KIIQPDKNNY AAMVFHYMSI TILVFFMMEI IFKLFVFRL-
ciHv1    ---------L KVIIVPHGNP APEILHGFSL SILSIFMVEI ALKIIADHR-
npHv1    --------LE MH---EEESL AQHVLHYCSI TILSIFIVEI FLKLYAFRQ-
etHv1    --------AS GVEKSEALET VEIALKFISI SILSIFVIEN LFTMYVLRC-
spHv1    ---EGQCNAT ETDKEEKEVT AANVLHYISI GILSIFMIEL LIKIPVFRM-
acHv1    --------LE IVKLGEEHHY IPKIFHYGSL GILSLFLIEI GLRIFVLRL-
acHv2    MYGNDSAHAA PVHHHTNKEK AEHVLHALSL TILSIFMVEV CVKIYVEGK-
cgHv4    ---------- ---EEHLREE LEDGLHYAAL TIISLFVVEV VIKIYIEGK-
aoHv1    --------KH GSHVAIGWGV TQKVLAIVGL VFSCLFMLEL MVTVFSFGK-
amHv1    ---------L RAGSEHHDNS ASHVLHYISI AILSVFMIEL LLKIYAMGF-
kvHv1    -----DPSHL AHYGNHDLHE WAERMEYASL AILLIFLLEN MLLVLANGC-
kvHv2    ----GECAGH PHFGDHTLHD IEIILAYISV GILSLFLVEQ VLLIVDLGK-
ehHv1    TVEGPHGVGC DEHAHPAVHT AHAVLTWASV AILSLFEIEL LTLLAASGLR
ehHv2    HLDSTGRIGC DSHKHDLAHK FHKFLFRVSL TVLVVFELEL LGLIASLDR-
egHv1    ----QTCHYN HPSLATALHL AEKVLAYISI GLLSFFLLER LVGIICEGL-
```

```
             ....|....| ....|....| ....|....| ....|....| ....|....|
                    310        320        330        340        350
hsHv1        EFFHHKFEIL DAVVVVVSFI LDIVLLFQ-- -EHQFEALGL LILLRLWRVA
ciHv1        HFIHHKVEVL DAVVVVISFG VDIALIFVGE -SEALAAIGL LVILRLWRVF
npHv1        EFFKHRLEVF DAIIVIVSFA LDIAFRNS-- -RDALSGVGL IIILRLWRVA
etHv1        EFFHKCLEVF DSIVIFTSLV LEVIFLNH-- -HDAATGVGI LIGLRLWRIV
spHv1        EFFRSKLEVF DGIIIVISFV LDVVSLIY-- -EEQFAVLQL LVLLRLWRIV
acHv1        DFFKHKLELF DAVVVIVSFI LDIVFRDN-- -EDAATGVGL LIILRLWRVT
acHv2        HMLKQKAEVF DAIVVIVSFT LDITFSFVSV SKAASEAAGL MVILRLWRVT
cgHv4        HFFSDKWEVF DAIVIFVTFG LDLALAFSPV SSAVRDSVAL LVFLRLWRVV
aoHv1        GYFSSKFHVF DALVIIVAFG VDVALHGI-- ---EEELGSL IVVLRLWRVF
amHv1        TFFKHKMEVF DGFVIIVSFA LDIAFSNE-- -QGGVDGVSL IVLLRLWRVT
kvHv1        RFFANPFHIL DIVVVVVSVG FELQGILG-- -EGHDAGIGL VVFARTWRFI
kvHv2        -EYLKPMFIL DFVVIVSSLL IEILVVNM-- -----TIGGL LVLARTWRFA
ehHv1        DFFSNVYYVL DIVIVSASLV LECVFYNT-- -AGLSDLIGL VMFLRLWRLL
ehHv2        AFFRNPLYVL DLIVITVSLG LETVFRVFS- -MPEQDLAMA LIIVRLWRFV
egHv1        KFFSCAFKVF DFVVIIVSLV LEILFLGQ-- -----PGVGL IAIGRFWRFV

             ....|....| ....|....| ....|....| ....|....| ....|....|
                    360        370        380        390        400
hsHv1        RIINGIIISV KTRSERQLLR LKQMNVQLAA KIQHLEFS-C SEKEQEIERL
ciHv1        RIINGIIVTV KTKADDRVHE IKKKNSELEL QIHNLEEK-L SQKEQDMSRL
npHv1        RVLNGVVLSV KMQAEHQLER EKQRGMALEG ELSRCRQV-C AAQQRELDVL
etHv1        RVINGIAVTV AAQSEKKLEE EKDLREEIEM ELNKCVDR-S KKQQCYILFL
spHv1        RVVNGVILSV ETQAKKKIEQ QKHLRAEVEH EMEKFRRY-C AAQEKEIEVL
acHv1        RIVNGIVLSV QKQAEKKIER EKHLREECEQ ELAKFREY-C MAQEAEIEVL
acHv2        RIINGVIMSV KLDANKKMEV HKKARRKLER ENKRLQAK-I ERLEREVATL
cgHv4        KIMTEIQYCV RREVPEAIDK EKLAQEKADW LARRQGHT-K V---------
aoHv1        KIIEELQSAN EDTLEEYEHE IERLRQENTY LRQRLNVS-L SNADPMD---
amHv1        RIVNGIILSV QMRAEKKVQA VMKENAELQK ELEQLKSK-C AQLESELTTL
kvHv1        RLGHGIHEMH EEHEAEDHGE HRVSDAAGSL EAPLQKGS-F EQHAKGTSGV
kvHv2        RVGHGVFEAK EMIEEMIGED DDTVKNMMDA WKELTPERWE EILHSGSAEI
ehHv1        RIGHAMFAST ERASSTDNLK EVVRELRAEL DLLSEWAE-- ---EEERASA
ehHv2        RIGHGIFAST HSVAKEKSDK LHAEIRALEE EYTRALNQAA SLATTTRPTR
egHv1        RIGHGFHEAE ELTHPVDEFV KEHKAALFAL HRRLLGVPGL YGTEGAVLAE

             ....|....| ....|....| ....|....| ....|....| ....|....|
                    410        420        430        440        450
hsHv1        NKLLRQHGLL GEVN------ ---------- ---------- ----------
ciHv1        HEILRCNNID IPPTVPLTTS VQIHSTTTAS ADV------- ----------
npHv1        RAVLQHHGLD QQLPDGNR-- ------VDVV ADVEKR---- ----------
etHv1        QKFIQSNGLE IPPHSSCPSR ---------- ---NSSEGH- ----------
spHv1        RNTLNQHGIQ IDDDYVAKKP QFSLNQLNVV VEMNSADKHD TGEDEGEGEE
acHv1        QGLLHKHNIE FTTNKITRPE --SRVQVDVV AEVNSMTAVA ETDIPLSPSQ
acHv2        KQKMATSSTP QMSFEMQSGL SVERSPSGEM RENSAQV--- ----------
amHv1        KQS------- ---------- ---------- ---------- ----------
kvHv1        HHARSQASSN REGREGCCVQ ---------- ---------- ----------
kvHv2        LRESGVTPAE IKLGEALGNS PAVAMRALAF ARGWKQKLDK KKAKRLSGSN
ehHv1        RAPPDDPGVD DIG------- ---------- ---------- ----------
ehHv2        LASAPSSTSG SPSATRPPPF RGRSASEASA ATHDLQGAGG EKLADGVAAA
egHv1        NAAAVREALA LLEQDGFGGL LLGIADAAVA GHRQHQEKEQ LHYWKLQ---

             ....|....| ....|....| ....|....| ....|....| ....|....|
```

```
              460       470       480       490       500
spHv1   GGGDGNTRRH EKEREALGEH TITLTTDDNV NTIQADYHPQ DTTFT-----
acHv1   PGEQEISLSS GDNVTDAVVV V--------- ---------- ----------
kvHv2   PKRQMPGASL QTTATVGKGH SEH------- ---------- ----------
ehHv2   LSQRSNTPTS PVGAPAMRRP PAAAPLGAAH RALQLGPSSS AARTGMPGVG


        ....|....| ....|....| ....|....| ....|....| ....|
              510       520       530       540
ehHv2   TRMGAGEAAA AERQAPPAAE PEPAEGAAAA ESVELELDAL DMST-
```

Hv sequences were used from *Homo sapiens* (hsHv1, NM_001040107, Ramsey et al. 2006), *Ciona intestinalis* (ciHv1, NM_001078469, Sasaki et al. 2006), *Nicoletia phytophila* (npHv1, KT780722, Chaves et al. 2016), *Extatosoma tiaratum* (etHv1, GAWG01024136, Chaves et al. 2022), *Strongylocentrotus purpuratus* (spHv1, XM_030990962, Sakata et al. 2016), *Aplysia californica* (acHv1 & acHv2, XM_005100609 & XM_005093050, Chaves et al. 2023a), *Crassostrea gigas* (cgHv1, XM_011429833, Chaves et al. 2023b), *Aspergillus oryzae* (aoHv1, XM_001825513, Zhao & Tombola 2021), *Acropora millepora* (amHv1, MZ029046, Rangel-Yescas et al. 2021), *Karlodinium veneficum* (kvHv1 & kvHv2, JN255155 & HBOS01067516, Smith et al. 2011), *Emiliania huxleyi* (ehHv1 & ehHv2, HBNU01018021 & GIZZ01010784, Taylor et al. 2011), *Euglena gracilis* (egHv1, GDJR01054876). Transmembrane regions are shown in yellow, the totally conserved tryptophan residue within S4 in red, selectivity filter residues in light (D) or dark (E)purple, voltage-sensor positively charged residues in dark (R) or light (K)blue.
